# APOE4-promoted gliosis and degeneration in tauopathy are ameliorated by pharmacological inhibition of HMGB1 release

**DOI:** 10.1016/j.celrep.2023.113252

**Published:** 2023-10-19

**Authors:** Nicole Koutsodendris, Jessica Blumenfeld, Ayushi Agrawal, Michela Traglia, Oscar Yip, Antara Rao, Min Joo Kim, Maxine R. Nelson, Yung-Hua Wang, Brian Grone, Yanxia Hao, Reuben Thomas, Misha Zilberter, Seo Yeon Yoon, Patrick Arriola, Yadong Huang

**Affiliations:** 1 Gladstone Institute of Neurological Disease, Gladstone Institutes, San Francisco, CA 94158, USA; 2 Developmental and Stem Cell Biology Graduate Program, University of California, San Francisco, San Francisco, CA 94143, USA; 3 Neuroscience Graduate Program, University of California, San Francisco, San Francisco, CA 94158, USA; 4 Gladstone Institute of Data Science and Biotechnology, Gladstone Institutes, San Francisco, CA 94158, USA; 5 Biomedical Sciences Graduate Program, University of California, San Francisco, San Francisco, CA 94143, USA; 6 Gladstone Center for Translational Advancement, Gladstone Institutes, San Francisco, CA 94158, USA; 7 Departments of Neurology and Pathology, University of California, San Francisco, San Francisco, CA 94143, USA; 8 Lead contact

## Abstract

Apolipoprotein E4 (APOE4) is an important driver of Tau pathology, gliosis, and degeneration in Alzheimer’s disease (AD). Still, the mechanisms underlying these APOE4-driven pathological effects remain elusive. Here, we report in a tauopathy mouse model that APOE4 promoted the nucleocytoplasmic translocation and release of high-mobility group box 1 (HMGB1) from hippocampal neurons, which correlated with the severity of hippocampal microgliosis and degeneration. Injection of HMGB1 into the hippocampus of young APOE4-tauopathy mice induced considerable and persistent gliosis. Selective removal of neuronal APOE4 reduced HMGB1 translocation and release. Treatment of APOE4-tauopathy mice with HMGB1 inhibitors effectively blocked the intraneuronal translocation and release of HMGB1 and ameliorated the development of APOE4-driven gliosis, Tau pathology, neurodegeneration, and myelin deficits. Single-nucleus RNA sequencing revealed that treatment with HMGB1 inhibitors diminished disease-associated and enriched disease-protective subpopulations of neurons, microglia, and astrocytes in APOE4-tauopathy mice. Thus, HMGB1 inhibitors represent a promising approach for treating APOE4-related AD.

## In brief

Nicole Koutsodendris et al. report that, in a tauopathy mouse model, APOE4 promoted intraneuronal translocation and release of HMGB1, which correlated with the severity of microgliosis and neurodegeneration. Treatment of APOE4-tauopathy mice with HMGB1 inhibitors effectively blocked the intraneuronal translocation and release of HMGB1 and ameliorated APOE4-driven Alzheimer’s disease pathologies.

## Graphical Abstract

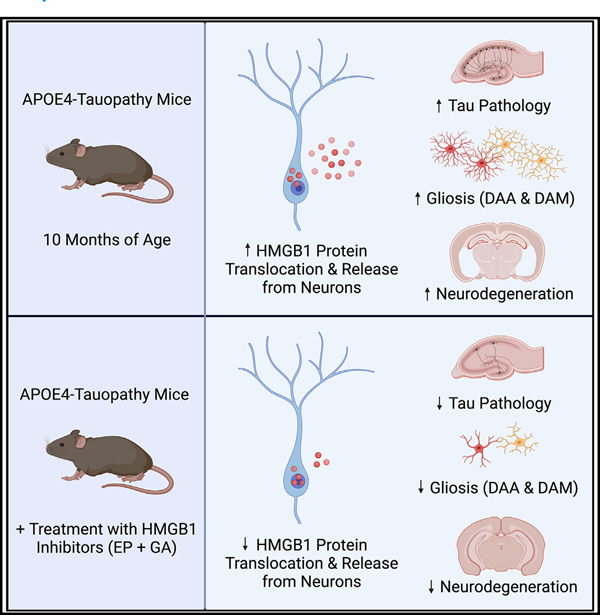

## INTRODUCTION

Alzheimer’s disease (AD) is a highly prevalent neurodegenerative disorder that involves progressive memory loss and cognitive decline.^1^ Currently, there are no viable therapeutic options to slow AD progression,^2^ which is likely due to an incomplete understanding of its pathogenic mechanisms. AD is classified as a major tauopathy disorder since one of its main pathological hallmarks is the accumulation of Tau protein aggregates within neurons.^3–5^ Other important AD pathological hallmarks include neuroinflammation and gliosis, which have recently been identified as major contributing factors to neurodegeneration in AD.^6,7^

Apolipoprotein E4 (*APOE4*) has been identified as the strongest genetic risk factor for late-onset AD.^8–10^ The human *APOE* gene exists as three common alleles, comprising ε2, ε3, and ε4. *APOE* ε4 is considered the most detrimental allele since it dose-dependently increases AD risk and decreases the age of disease onset.^8–11^ There is considerable evidence supporting the notion that APOE4 and APOE3 have vastly different effects on AD pathogenesis,^1,12–14^ with APOE4 worsening Aβ fibrillization and clearance,^15–17^ Tau phosphorylation and aggregation,^18–23^ and glial dysfunction^14,24^ relative to APOE3. In addition, APOE4 accelerates hippocampal volume loss^25^ and reduces myelination^26^ in human patients and increases neurodegeneration in mice with^22^ or without tauopathy^27–29^ relative to APOE3. Moreover, recent studies have shown that APOE4-promoted gliosis^22,23,30^ is a strong driver of neurodegeneration,^7^ although the underlying cellular and molecular mechanisms are unclear.

High-mobility group box 1 (HMGB1) is a ubiquitous nuclear protein^31^ that mediates pathogenic inflammatory responses in a variety of neurodegenerative disorders, including epilepsy,^32^ traumatic brain injury,^33^ Parkinson’s disease,^34^ and multiple sclerosis.^35^ Under physiological conditions, HMGB1 is localized to the nucleus and it operates as a transcriptional regulator.^36,37^ Under pathological conditions, HMGB1 translocates from the nucleus to the cytoplasm of the cell,^38,39^ where it functions as an autophagy driver and nucleic acid sensor.^31^ HMGB1 is then released to act as a key damage-associated molecular pattern (DAMP)^40^ that activates immune cells by binding with its receptors and activating the NF-κB pathway,^41,42^ thus promoting inflammation.^31,43–45^ The role of HMGB1 translocation and release in AD pathogenesis is still unclear and understudied, although some studies have shown that HMGB1 levels are elevated in the cerebrospinal fluid (CSF) of human AD patients^46^ and that Aβ^46^ and Tau^47^ oligomers can induce HMGB1 translocation and release from some brain cells.

While previous studies suggest a role for HMGB1 in AD, there is an evident gap in knowledge as to whether HMGB1 is a key player in the mechanisms of APOE4-driven AD pathogenesis. As 60%–75% of late-onset AD patients are APOE4 carriers,^11,48^ it is critically important to study the potential relationship between APOE4 and HMGB1 in AD pathogenesis. In the current study, we aimed to gain an in-depth understanding of the relationship between APOE4 and HMGB1 in the setting of tauopathy by (1) evaluating the differential effects of APOE4 and APOE3 on the nucleocytoplasmic translocation and release of HMGB1 protein; (2) elucidating the potential APOE isoform-specific connection between HMGB1 translocation and release, gliosis, and degeneration; and (3) determining the therapeutic efficacy of utilizing HMGB1 inhibitors to mitigate the development of APOE4-driven AD pathologies in a tauopathy mouse model. Ultimately, the goal of the study is to understand the role of HMGB1 in APOE4-driven AD pathogenesis and to identify new therapeutic approaches combating APOE4-related AD and other tauopathies.

## RESULTS

### APOE4 exacerbates degeneration and gliosis in a mouse model of tauopathy

To investigate the relationship between APOE4 and HMGB1 in the setting of tauopathy, we utilized a compound mouse model with humanized APOE and transgenic Tau-P301S expression. To generate this mouse model, the homozygous human *APOE4* or *APOE3* knockin mice previously generated in our lab^49^ were cross-bred with a widely utilized tauopathy mouse model (PS19 line) that expresses P301S mutant human microtubule-associated protein Tau (*MAPT*).^50^ The resulting PS19-E4 and PS19-E3 mice were used in this study.

We first performed an in-depth characterization of the pathological differences between 10-month-old PS19-E4 and PS19-E3 mice. The PS19-E4 mice displayed extensive neurodegeneration relative to PS19-E3 mice, as they had a significant decrease in hippocampal volume and increase in posterior lateral ventricle volume (Figures S1A–S1C). PS19-E4 mice also had more pronounced hippocampal Tau pathology relative to PS19-E3 mice (Figures S1D and S1E). We then performed sequential biochemical extraction of mouse hippocampal lysates to separate highly soluble and less soluble Tau proteins in the RAB and RIPA buffer fractions, respectively. Western blot analysis revealed that PS19-E4 mice had a minor increase in soluble AT8^+^ p-Tau in the RAB fraction and a significant increase in less soluble p-Tau in the RIPA fraction relative to PS19-E3 mice (Figures S2A–S2D). Immunohistochemical analysis of myelin basic protein (MBP) in the stratum radiatum of CA1 showed that PS19-E4 mice had a significantly lower coverage area of MBP relative to PS19-E3 mice (Figures S1F and S1G), indicating that APOE4 leads to hippocampal myelin deficits in the context of tauopathy.

Assessment of glial cells revealed that PS19-E4 mice had significantly higher hippocampal coverage areas of Iba1^+^ microglia (Figures S1H and S1I) and GFAP^+^ astrocytes than PS19-E3 mice (Figures S1J and S1K). Immunostaining for markers of activated glial cells showed that PS19-E4 mice had significantly higher hippocampal coverage of CD68^+^ activated microglia^51^ (Figures S2E and S2F) and S100β^+^ activated astrocytes^52^ than PS19-E3 mice (Figures S2G and S2H). These data suggest that APOE4 promotes hippocampal microgliosis and astrogliosis in this tauopathy mouse model. There was a strong negative correlation between microglia (Figure S1L) or activated microglia (Figure S2I) coverage area and hippocampal volume in PS19-E4 mice. There was also a weak, but significant, negative correlation between astrocyte (Figure S1M) or activated astrocyte (Figure S2J) coverage area and hippocampal volume in PS19-E4 mice. These data indicate that the extents of gliosis and neurodegeneration are tightly related and suggest that microgliosis might be a better indicator and/or contributor to neurodegeneration than astrogliosis. Taken together, this detailed pathological characterization shows that APOE4 has wide-ranging detrimental effects that exacerbate various AD-related pathologies and is complementary to what has been previously reported.^22,23,53,54^

### APOE4 promotes the nucleocytoplasmic translocation of HMGB1 in hippocampal neurons

**Figure 1.**
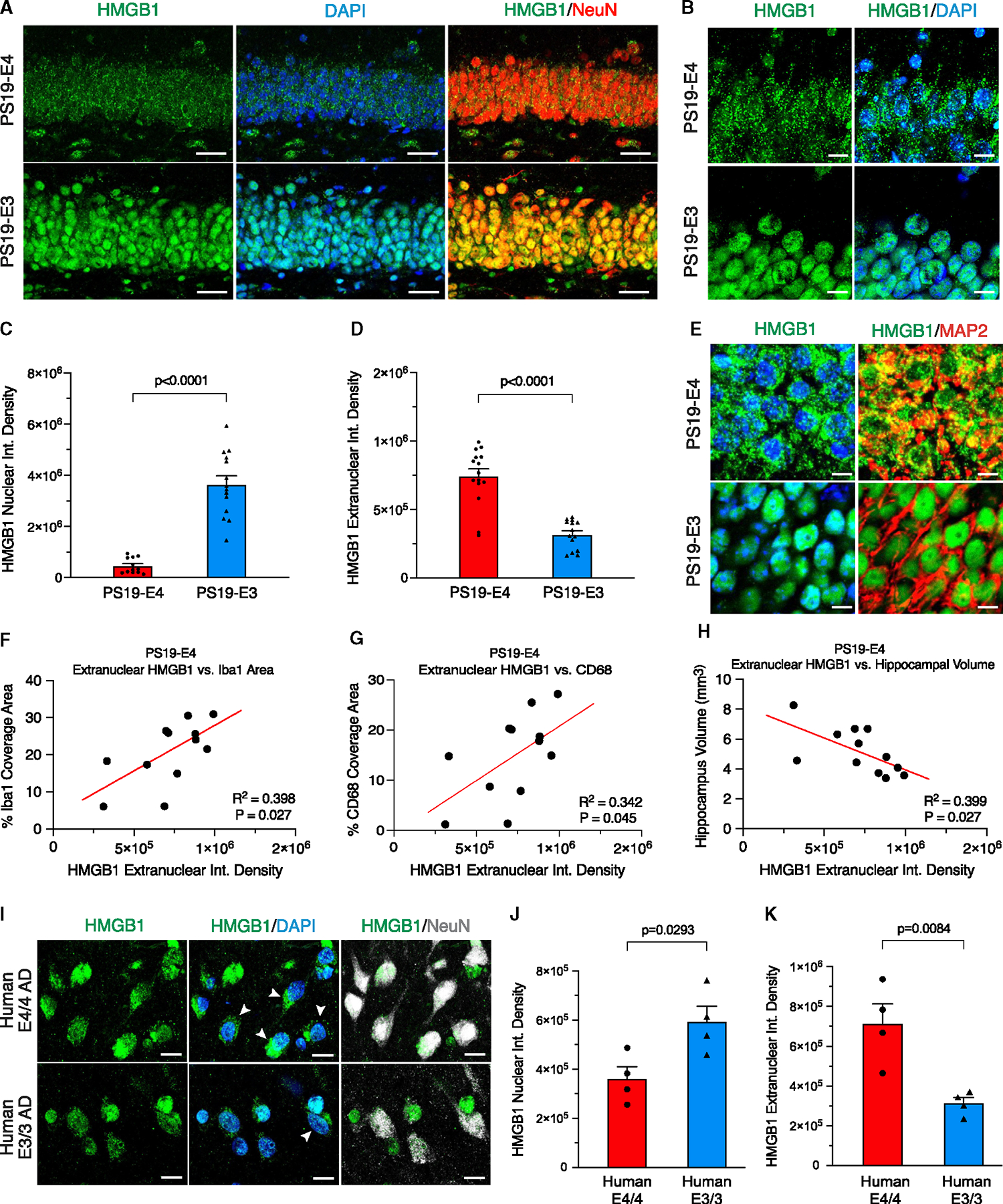
APOE4 promotes the nucleocytoplasmic translocation of HMGB1 in hippocampal neurons in tauopathy mice (A) Representative images of immunostaining with anti-HMGB1, anti-NeuN, and DAPI in the dentate gyrus of the hippocampus (scale bar, 40 μm). (B) Representative high-magnification images of immunostaining with anti-HMGB1 and DAPI in the dentate gyrus of the hippocampus (scale bar, 10 μm). (C and D) Quantification of the nuclear integrated density (C) and extranuclear integrated density (D) of HMGB1 immunostaining in hippocampal neurons (E) Representative images of immunostaining with anti-HMGB1, anti-MAP2, and DAPI in the dentate gyrus of the hippocampus in PS19-E4 and PS19-E3 mice (scale bar, 5 μm). (F) Correlation between HMGB1 extranuclear integrated density in neurons and the percentage of Iba1 coverage area. (G) Correlation between HMGB1 extranuclear integrated density in neurons and the percentage of CD68 coverage area. (H) Correlation between HMGB1 extranuclear integrated density in neurons and hippocampal volume. (I) Representative images of immunostaining with anti-HMGB1, DAPI, and NeuN in the cortex of human AD patient brain samples with different APOE genotypes (scale bar, 5 μm). Arrowheads indicate neurons (NeuN positive) with HMGB1 translocation into the cytoplasm. (J) Quantification of the nuclear integrated density of HMGB1 immunostaining in neurons in human AD brains with different APOE genotypes. (K) Quantification of the extranuclear integrated density of HMGB1 immunostaining in neurons in human AD brains with different APOE genotypes. For all representative images and quantified data in (A)–(H), mice were 10 months of age and belonged to either the PS19-E4 or the PS19-E3 group. Quantified data in (C) and (D) (PS19-E4, n = 12; PS19-E3, n = 14) and (J) and (K) (human E4/4 AD, n = 4; human E3/3 AD, n = 4) are represented as the mean ± SEM, unpaired two-tailed t test. Data in (F), (G), and (H) (PS19-E4, n = 12) are Pearson’s correlation analysis (two-tailed). For demographic information of samples used in (I)–(K), see Table S1.

HMGB1 is a nuclear protein widely recognized as a critical factor in glial cell activation.^39^ Under pathological conditions, HMGB1 translocates from the nucleus to the cytoplasm of stressed or dying cells and is then released to act as a proinflammatory DAMP to activate glial cells.^36,38,44^ Since gliosis is one of the earliest pathological manifestations in the PS19 tauopathy mouse model^50^ and APOE4 promotes gliosis in this same mouse model (Figures S1H–S1K and S2E–S2H), we investigated if APOE4 affects the nucleocytoplasmic translocation of HMGB1 that can trigger glial response. Immunohistochemical staining for HMGB1 and the nuclear marker DAPI in 10-month-old mice showed that PS19-E4 mice had a remarkably high amount of nucleocytoplasmic translocation of HMGB1, as a significant majority of it was located outside of the nucleus in hippocampal neurons (Figures 1A–1D), some of which was colocalized with MAP2 (Figure 1E). Conversely, PS19-E3 mice exhibited significantly lower levels of HMGB1 nucleocytoplasmic translocation in hippocampal neurons (Figures 1A–1E).

To investigate whether APOE4 promotes HMGB1 translocation in the absence of tauopathy, we analyzed human APOE (E) knockin mice that lacked human mutant Tau-P301S.^49^ Immunostaining for HMGB1 in 10-month-old mice showed that the majority of HMGB1 remained localized within the nucleus of hippocampal neurons in both E4 and E3 mice (Figures S3A–S3D). This illustrates that HMGB1 translocation requires the coexistence of both APOE4 and tauopathy, as observed in the PS19-E4 mice (Figures 1A–1E). Notably, there was no significant difference in hippocampal volume, Tau pathology, or myelin deficits between E4 and E3 knockin mice, although a minor difference in gliosis between these genotype groups was observed (Figures S3E–S3N).

To further dissect the relationship between HMGB1, APOE genotype, tauopathy, and aging, we assessed the extent of neuronal HMGB1 translocation in 6-month-old PS19-E4 and PS19-E3 mice. Immunostaining for HMGB1 protein illustrated that, unlike the 10-month-old PS19-E mice (Figures 1A–1E), there was no significant difference in HMGB1 translocation in hippocampal neurons between 6-month-old PS19-E4 and PS19-E3 mice (Figures S4A–S4C). Examining the extent of gliosis in these younger mice showed that there was also no significant difference in the coverage area of microglia or astrocytes in the hippocampus of 6-month-old PS19-E4 and PS19-E3 mice (Figures S4D–S4F). Analysis of the correlation between HMGB1 translocation with gliosis in the time axis between 6- and 10-month-old PS19-E4 mice revealed that there was a significant correlation between the relative amounts of extranuclear HMGB1 in neurons and the percentage of coverage areas of microglia and astrocytes in the hippocampus across age in PS19-E4 mice (Figures S4G and S4H). These data indicate that aging is also an important factor in APOE4-promoted HMGB1 translocation and provide evidence that there is a relationship between HMGB1 translocation and gliosis in PS19-E4 mice.

We also analyzed the relationship between HMGB1 translocation, gliosis, and neurodegeneration within the aged, 10-month-old PS19-E mice. In PS19-E4 mice, the relative amount of extranuclear HMGB1 in neurons had significant positive correlations with the coverage areas of microglia (Figure 1F) and activated microglia (Figure 1G), a significant negative correlation with hippocampal volume (Figure 1H), but no correlation with the coverage areas of astrocytes (Figure S4K) or activated astrocytes (Figure S4L). Such correlations were not observed in PS19-E3 mice (Figures S4M–S4Q). Together, these data revealed an intriguing relationship between the extent of neuronal HMGB1 translocation and the severity of microgliosis and neurodegeneration in the presence of APOE4, suggesting that HMGB1 likely plays an important role in the pathogenic mechanism of APOE4-driven gliosis and consequently neurodegeneration in the context of tauopathy.

Since APOE is also expressed in astrocytes^55^ and microglia,^56,57^ we then examined whether HMGB1 translocation occurs in these glial cell types in 10-month-old PS19-E mice. We observed that PS19-E4 mice had a minor, yet significant, increase in HMGB1 translocation in astrocytes, while displaying no significant increase in HMGB1 translocation in microglia, relative to PS19-E3 mice (Figures S5A–S5F). Unlike neuronal HMGB1 (Figure 1H), there was no significant correlation between hippocampal volume and the relative amounts of extranuclear HMGB1 protein in astrocytes or microglia in PS19-E4 mice (Figures S5G and S5H). Comparison of the ratio of extranuclear: nuclear HMGB1 fluorescence intensity between neurons and these glial cells in PS19-E4 mice revealed that neurons exhibit a significantly higher proportion of extranuclear HMGB1 compared to astrocytes and microglia (Figure S5I). Overall, these data indicate that HMGB1 nucleocytoplasmic translocation is much more pronounced in hippocampal neurons than in astrocytes in PS19-E4 mice, suggesting a greater role of neuronal HMGB1 translocation in contributing to downstream HMGB1-induced pathologies.

We next examined whether APOE4 promotes intracellular translocation of HMGB1 in human brains to validate our observations in tauopathy mouse models. We analyzed the prefrontal cortex samples of eight AD patients from the ROSMAP cohort,^58^ four of whom were homozygous for APOE4, and the other four of whom were homozygous for APOE3. All eight AD patients presented with a Cogdx score of 4 (Table S1). Immunohistochemical staining for HMGB1 illustrated that APOE4/4 AD patients have significantly higher HMGB1 translocation in neurons compared with APOE3/3 AD patients (Figures 1I–1K). This finding validates the observations in PS19-E4 mice and provides evidence that APOE4 promotes neuronal HMGB1 translocation in a human and disease-relevant context.

### APOE4-driven release of HMGB1 induces acute and persistent gliosis in the hippocampus

**Figure 2.**
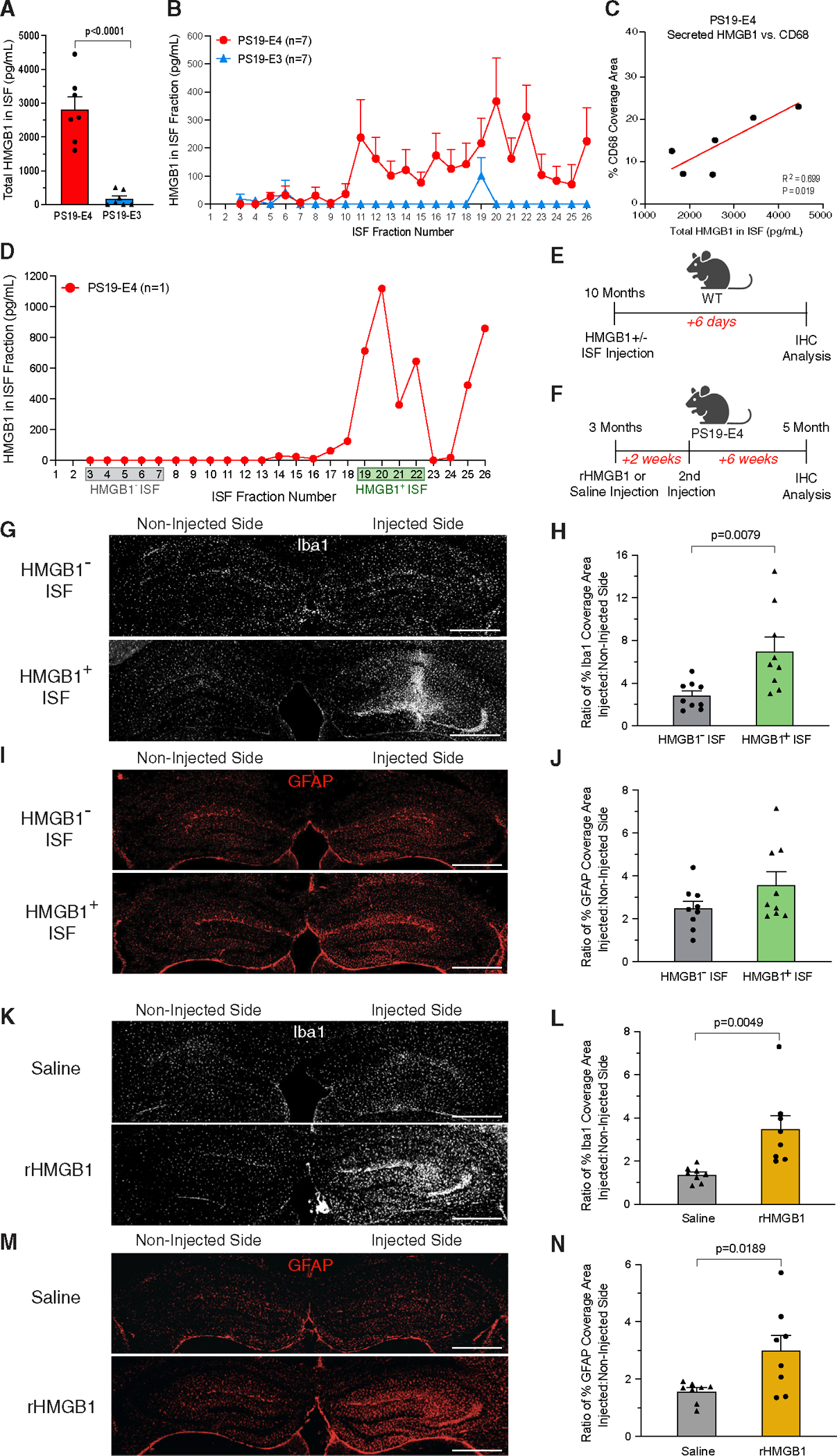
APOE4 promotes the cellular release of HMGB1 to induce acute and persistent gliosis in mouse hippocampus (A) HMGB1 protein levels measured by ELISA in the hippocampal interstitial fluid (ISF) of 10-month-old mice. (B) HMGB1 protein levels measured by ELISA in each collected ISF fraction of 10-month-old PS19-E4 mice. Fractions 1 and 2 were excluded from analyses in (A) and (B) since artificial CSF was circulated at a higher flow rate for the first 2 h to prevent clogging of the tubing. (C) Correlation between HMGB1 protein levels in the ISF and the percentage of CD68 coverage area in 10-month-old PS19-E4 mice. (D) HMGB1 protein levels measured by ELISA in each collected ISF fraction of one PS19-E4 mouse used for experiments in (E) and (G)–(J). Fractions 3–7 were designated as HMGB1 absent (HMGB1^−^) and fractions 19–22 were designated as HMGB1 enriched (HMGB1^+^). (E) Experimental design of a study involving the injection of HMGB1-enriched or HMGB1-absent ISF into the hippocampus of 10-month-old wild-type (WT) mice and assessment of acute gliosis 6 days post-injection. (F) Experimental design of a study involving two injections of recombinant HMGB1 (rHMGB1) or saline into the hippocampus of 3-month-old PS19-E4 mice and assessment of gliosis 8 week post-injection. (G) Representative images of microglia stained with anti-Iba1 in the hippocampus of 10-month-old WT mice, part of study in (E) (scale bar, 500 μm). (H) Quantification of the ratio of the percentage of Iba1 coverage area between the injected:non-injected hippocampal sides 6 days post-injection. (I) Representative images of astrocytes stained with anti-GFAP in the hippocampus of 10-month-old WT mice, part of study in (E) (scale bar, 500 μm). (J) Quantification of the ratio of the percentage of GFAP coverage area between the injected:non-injected hippocampal sides 6 days post-injection. (K) Representative images of microglia stained with anti-Iba1 in the hippocampus of PS19-E4 mice, part of study in (F) (scale bar, 500 μm). (L) Quantification of the ratio of the percentage of Iba1 coverage area between the injected:non-injected hippocampal sides 8 week post-injection. (M) Representative images of astrocytes stained with anti-GFAP in the hippocampus of PS19-E4 mice, part of study in (F) (scale bar, 500 μm). (N) Quantification of the ratio of the percentage of GFAP coverage area between the injected:non-injected hippocampal sides 8 week post-injection. In (A) and (B), n = 7 mice per genotype. In (C) (PS19-E4, n = 12), Pearson’s correlation analysis (two-tailed). In (H) and (J), n = 9 mice for control (HMGB1^−^) and experimental (HMGB1^+^) groups. In (L) and (N), n = 8 mice for rHMGB1 and saline control groups. All data are represented as the mean ± SEM, unpaired two-tailed t test.

To further evaluate the differential effects of APOE4 and APOE3 on the cellular release of HMGB1 protein, we collected the hippocampal interstitial fluid (ISF) from 10-month-old PS19-E mice over a 24-h period using *in vivo* microdialysis.^59^ We quantitatively determined the levels of extracellular HMGB1 protein in the ISF using sandwich ELISA. Strikingly, PS19-E4 mice had much higher levels of HMGB1 in their hippocampal ISF than PS19-E3 mice (Figures 2A and 2B), indicating that APOE4 strongly promotes the cellular release of HMGB1 in the hippocampus. After staining brain sections from the PS19-E4 mice utilized for ISF collection, we observed a strong correlation between the levels of HMGB1 in hippocampal ISF and the coverage area of activated microglia in the hippocampus (Figure 2C), suggesting a relationship between cellular HMGB1 release and activation of microglia in the hippocampus.

It has been reported that, following its cellular release, HMGB1 acts as a pro-neuroinflammatory DAMP that induces gliosis.^44,60^ To determine the importance of released HMGB1 in triggering acute gliosis in APOE4-related tauopathy, 10-month-old wild-type mice received a unilateral injection of hippocampal ISF collected from 10-month-old PS19-E4 mice that either was enriched with high concentrations of HMGB1 (fractions 19–22 in Figure 2D) or had undetectable levels of HMGB1 as a control (fractions 3–7 in Figure 2D). These mice were analyzed 6 days post-injection to assess acute changes in glial response in the hippocampus (Figure 2E). We quantified the ratio of glial coverage area on the injected hippocampal side to the non-injected side to control for the surgical procedure and to normalize for potential differences in glial cells on the non-injected side between mice.

Wild-type mice injected with the HMGB1-absent ISF exhibited relatively low coverage areas of microglia and astrocytes on the injected and non-injected hippocampal sides (Figures 2G–2J). In contrast, wild-type mice injected with the HMGB1-enriched ISF displayed a significant increase in the coverage area of microglia (Figures 2G and 2H) and a minor, yet insignificant, increase in the coverage area of astrocytes on the injected hippocampal side (Figures 2I and 2J). These data indicate that HMGB1-enriched ISF from PS19-E4 mice leads to a strong acute recruitment/activation of microglia, while showing only a mild increase in the extent of astrocyte recruitment/activation.

To determine whether HMGB1 can induce persistent gliosis after its release in the context of APOE4, we injected recombinant HMGB1 (rHMGB1) or saline directly into the hippocampus of young PS19-E4 mice that were 3 months of age (Figure 2F). These mice received a second injection 2 weeks after the initial injection to mimic the repeated cellular release of HMGB1. Immunohistochemical analysis 8 weeks post-injection showed that the mice injected with rHMGB1 had significantly higher levels of hippocampal microgliosis and astrogliosis on the injected side compared with saline-injected mice (Figures 2K–2N). This illustrates that HMGB1 is able to induce persistent gliosis in young PS19-E4 mice even several weeks post-injection and suggests that the HMGB1 release from hippocampal cells represents a mechanism by which APOE4 promotes gliosis in the context of tauopathy.

### APOE4 promotes acetylation of HMGB1 and decreases levels of SIRT1 deacetylase

**Figure 3.**
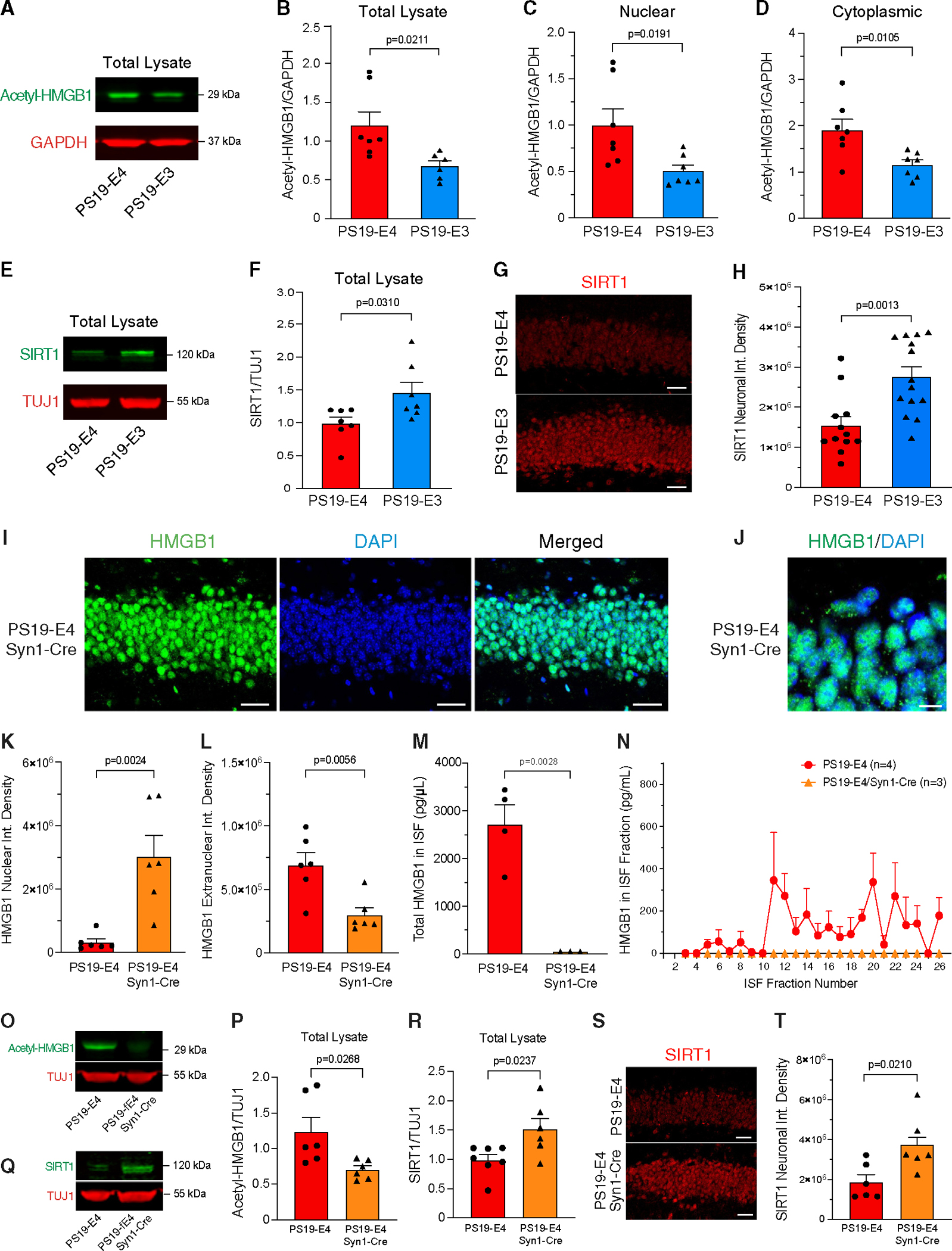
APOE4 promotes acetylation of HMGB1 and decreases levels of SIRT1 deacetylase, and removal of neuronal APOE4 reduces HMGB1 acetylation and increases SIRT1 levels (A) Representative images of anti-acetyl-HMGB1 (green) and anti-GAPDH (red) western blots in hippocampal tissue lysates. (B) Quantification of acetyl-HMGB1 levels relative to GAPDH. (C and D) Quantification of acetyl-HMGB1 levels relative to GAPDH in the nuclear fraction (C) and cytoplasmic fraction (D). (E) Representative images of anti-SIRT1 (green) and anti-TUJ1 (red) western blots in hippocampal tissue lysates. (F) Quantification of SIRT1 levels relative to TUJ1. (G) Representative images of neurons stained with anti-SIRT1 in the hippocampus (scale bar, 60 μm). (H) Quantification of the integrated density of SIRT1 in hippocampal neurons. (I) Representative images of immunostaining with anti-HMGB1 and DAPI in hippocampal neurons (scale bar, 40 μm). (J) Representative high-magnification images of immunostaining with anti-HMGB1 and DAPI in hippocampal neurons (scale bar, 10 μm). (K and L) Quantification of the nuclear integrated density (K) and extranuclear integrated density (L) of HMGB1 immunostaining in hippocampal neurons. (M and N) HMGB1 protein levels measured by ELISA in all hippocampal interstitial fluid (ISF) fractions (M) and in each collected ISF fraction (N). (O) Representative images of anti-acetyl-HMGB1 (green) and anti-TUJ1 (red) western blots in hippocampal tissue lysates. (P) Quantification of acetyl-HMGB1 levels relative to TUJ1. (Q) Representative images of anti-SIRT1 (green) and anti-TUJ1 (red) western blots in hippocampal tissue lysates. (R) Quantification of SIRT1 levels relative to TUJ1. (S) Representative images of neurons stained with anti-SIRT1 in hippocampus (scale bar, 60 μm). (T) Quantification of the integrated density of SIRT1 in hippocampal neurons. Data in (B), (C), (D), (F), (P), and (R) are quantified by western blot analysis of hippocampal tissue lysates. For all representative images and quantified data, mice were 10 months of age and belonged to PS19-E4, PS19-E3, or PS19-E4/Syn1-Cre group as indicated. Quantified data in (B), (C), (D), and (F) (PS19-E4, n = 7; PS19-E3, n = 7); in (H) (PS19-E4, n = 12; PS19-E3, n = 14); and in (K), (L), (P), (R), and (T) (PS19-E4, n = 6; PS19-E4/Syn1-Cre, n = 6) are represented as the mean ± SEM, unpaired two-tailed t test. Fractions 1 and 2 were excluded from analyses in (O) and (P). Quantified data in (M) and (N) (PS19-E4, n = 4; PS19-E4/Syn1-Cre, n = 3) are represented as the mean ± SEM, unpaired two-tailed t test.

Several studies have shown that HMGB1 translocation is regulated by its post-translational modifications, with the acetylation of HMGB1 triggering its translocation to the cytoplasm and promoting its subsequent release.^61–64^ We evaluated the levels of acetylated HMGB1 in the hippocampal lysates of 10-month-old PS19-E mice by western blot using an antibody that specifically recognizes HMGB1 acetylated at Lys-29. PS19-E4 mice had significantly higher acetyl-HMGB1 levels in their hippocampal lysates than PS19-E3 mice (Figures 3A and 3B). We also processed hippocampal tissues from 10-month-old PS19-E mice using a protein extraction kit to separate nuclear and cytoplasmic protein fractions and showed that PS19-E4 mice had significantly higher levels of acetyl-HMGB1 in both the nuclear and the cytoplasmic fractions than PS19-E3 mice (Figures 3C and 3D).

Sirtuin 1 (SIRT1) functions as a deacetylase and has been shown to directly interact with HMGB1,^65,66^ with repression or loss of SIRT1 leading to increased acetyl-HMGB1 and promoting its intracellular translocation and release.^67,68^ Analysis of hippocampal lysates by western blot showed that PS19-E4 mice had significantly lower levels of SIRT1 than PS19-E3 mice (Figures 3E and 3F). Likewise, immunohistochemical analysis of SIRT1 in hippocampal neurons showed that PS19-E4 mice had significantly lower levels of SIRT1 staining compared with PS19-E3 mice (Figures 3G and 3H). Taken together, these data provide a possible mechanism through which APOE4 promotes the intracellular translocation of HMGB1, whereby APOE4 reduces SIRT1 deacetylase protein levels and consequently leads to increased acetylation of HMGB1 and its subsequent nucleocytoplasmic translocation and release from cells.

### Neuronal APOE4 promotes the nucleocytoplasmic translocation and release of HMGB1

While APOE is produced by several cell types in the brain, previous studies from our lab have indicated that neuronal APOE4 exerts detrimental effects of promoting p-Tau accumulation,^18,19^ Aβ production,^18^ inhibitory neuronal loss,^69^ and learning and memory deficits.^69^ We recently found that the removal of neuronal APOE4 strongly protected against Tau-mediated gliosis and degeneration.^23^ To determine if neuronal APOE4 plays a key role in promoting HMGB1 translocation and release, we cross-bred PS19-E4 mice that had a *loxP*-floxed human APOE gene with mice carrying Cre recombinase driven under a neuron-specific Syn1 promoter (Syn1-Cre) to generate PS19-E4/Syn1-Cre mice. In PS19-E4/Syn1-Cre mice, the *APOE4* gene was selectively removed from neurons.^23,69^ Immunohistochemical staining of HMGB1 in 10-month-old PS19-E4/Syn1-Cre mice revealed that there was significantly less HMGB1 translocation in hippocampal neurons after neuronal APOE4 removal, with the majority of HMGB1 remaining localized in the nucleus (Figures 3I–3L).

Furthermore, we determined the effect of removing neuronal APOE4 on the cellular release of HMGB1 by performing *in vivo* microdialysis to collect the hippocampal ISF from 10-month-old PS19-E4/Syn1-Cre mice. Quantitative analysis of HMGB1 levels via ELISA showed that neuronal APOE4 removal prevented the release of HMGB1 protein into the ISF, as there were no detectable levels of HMGB1 protein in the ISF of PS19-E4/Syn1-Cre mice (Figures 3M and 3N). Taken together, these data indicate that the expression of APOE4 in neurons is a strong driver of HMGB1 intraneuronal translocation and subsequent release into the ISF in the hippocampus.

We next evaluated whether neuronal APOE4 removal leads to decreased HMGB1 translocation by altering HMGB1 acetylation. Analysis of hippocampal lysates of 10-month-old mice by western blot with an acetyl-HMGB1-specific antibody showed that PS19-E4/Syn1-Cre mice had significantly reduced acetyl-HMGB1 levels versus PS19-E4 mice (Figures 3O and 3P). Concurrently, PS19-E4/Syn1-Cre mice exhibited significantly increased levels of SIRT1 in their hippocampal lysates by western blot analysis (Figures 3Q and 3R), and in neurons by immunostaining (Figures 3S and 3T), compared with PS19-E4 mice. This illustrates that one possible mechanism by which neuronal APOE4 removal protects against HMGB1 translocation is by increasing SIRT1 deacetylase protein levels and decreasing acetylation of HMGB1, thereby blocking its subsequent nucleocytoplasmic translocation and release from cells.

Interestingly, removal of neuronal APOE4 in PS19-E4/Syn1-Cre mice also significantly reduced HMGB1 translocation in astrocytes (Figures S5J–S5L). There was no such effect observed in microglia of PS19-E4/Syn1-Cre versus PS19-E4 mice (Figures S5M–S5O). These data indicate that the promoted intra-astrocyte translocation of HMGB1 in PS19-E4 mice is likely a secondary effect in response to neuronal APOE4’s detrimental actions.

### Short-term treatment with HMGB1 inhibitors blocks APOE4-driven HMGB1 release

**Figure 4.**
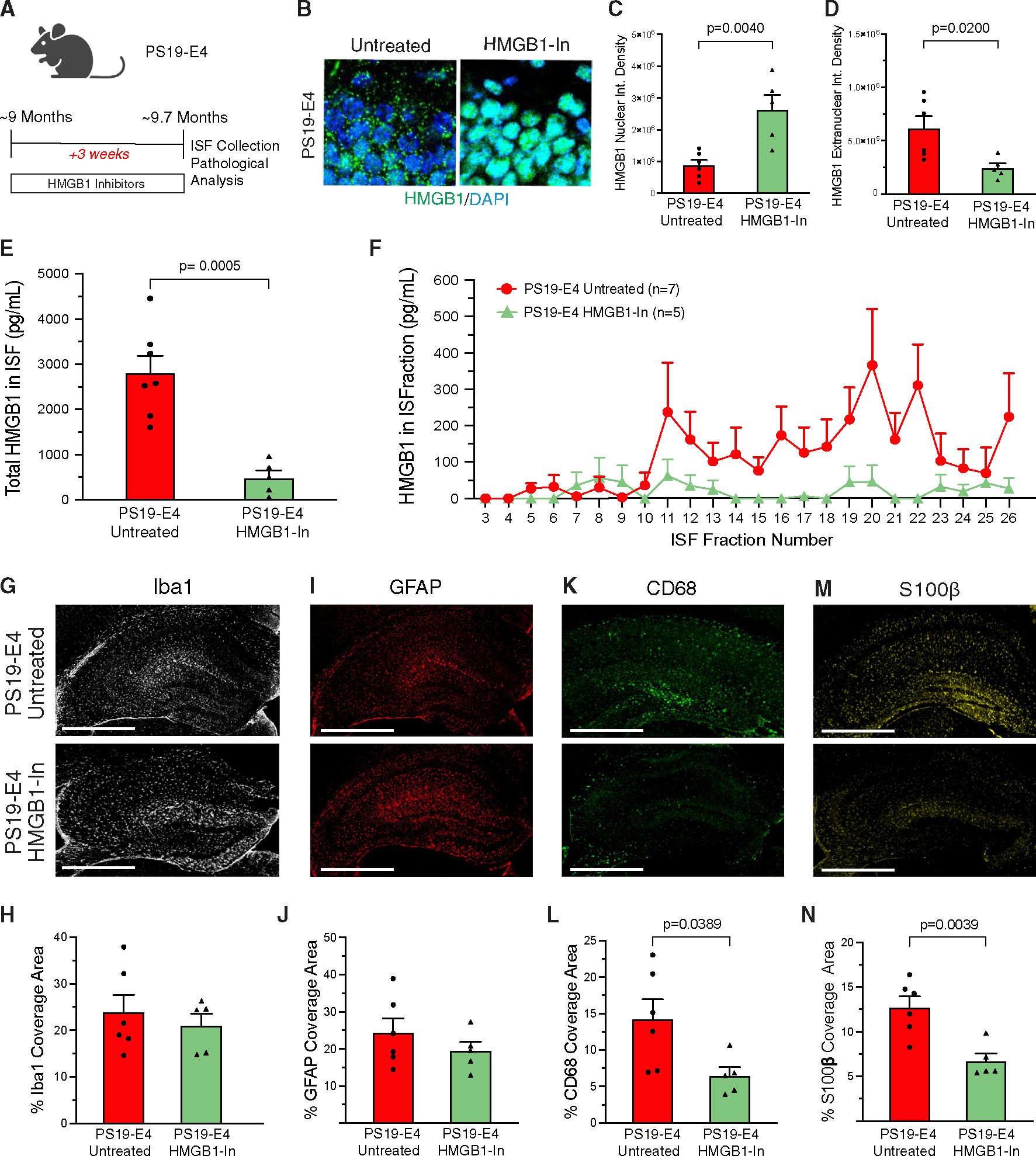
Short-term treatment of PS19-E4 mice with HMGB1 inhibitors blocks the nucleocytoplasmic translocation of HMGB1 in hippocampal neurons and its release into the ISF (A) Experimental design of the HMGB1 inhibitor study, with EP + GA treatment at three doses per week. (B) Representative high-magnification images of immunostaining with anti-HMGB1 and DAPI in hippocampal neurons (scale bar, 10 μm). (C and D) Quantification of the nuclear integrated density (C) and extranuclear integrated density (D) of HMGB1 immunostaining in hippocampal neurons. (E) HMGB1 protein levels measured by ELISA in the hippocampal interstitial fluid (ISF). (F) HMGB1 protein levels measured by ELISA in each collected ISF fraction. Fractions 1 and 2 were excluded from analyses in (E) and (F). (G) Representative images of microglia immunostaining with anti-Iba1 in the hippocampus (scale bar, 500 μm). (H) Quantification of the percentage of Iba1 coverage area in the hippocampus. (I) Representative images of astrocyte immunostaining with anti-GFAP in the hippocampus (scale bar, 500 μm). (J) Quantification of the percentage of GFAP coverage area in the hippocampus. (K) Representative images of activated microglia immunostaining with anti-CD68 in the hippocampus (scale bar, 500 μm). (L) Quantification of the percentage of CD68 coverage area in the hippocampus. (M) Representative images of activated astrocyte immunostaining with anti-S100β in the hippocampus (scale bar, 500 μm). (N) Quantification of the percentage of S100b coverage area in the hippocampus. For representative images and quantified data, mice were 9.7 months of age and belonged to either the untreated or the HMGB1 inhibitor-treated PS19-E4 group. Quantified data in (C), (D), (H), (J), (L), and (N) (PS19-E4 untreated, n = 6; PS19-E4 HMGB1-In, n = 5) are represented as the mean ± SEM, unpaired two-tailed t test. Quantified data in (E) and (F) (PS19-E4, n = 7; PS19-E4 HMGB1-In, n = 5) are represented as the mean ± SEM, unpaired two-tailed t test. HMGB1-In, HMGB1 inhibitors.

Based on our findings that APOE4 is a potent driver of HMGB1 intracellular translocation and release from neurons, we tested the therapeutic efficacy of HMGB1 inhibitors in combating APOE4-driven AD pathogenesis in the context of tauopathy. To this end, we utilized two well-characterized small molecules, ethyl pyruvate (EP) and glycyrrhizic acid (GA), which are established inhibitors of HMGB1 intracellular translocation and release.^70–73^ We first performed a short-term treatment to evaluate their ability to block HMGB1 intracellular translocation and release by administering a mixed solution of EP (80 mg/kg) and GA (20 mg/kg) to PS19-E4 mice at three doses per week for 3 weeks via intraperitoneal injections (Figure 4A).^59^ Treatment began when the mice were ~9 months of age, which is when the PS19-E4 mice exhibit pronounced pathology, and completed when the mice were ~9.7 months of age.

We first confirmed the effectiveness of EP and GA at blocking HMGB1 nucleocytoplasmic translocation in hippocampal neurons of 9.7-month-old mice after the short-term treatment. Untreated PS19-E4 mice exhibited extensive HMGB1 translocation out of the nucleus, whereas PS19-E4 mice treated with EP + GA for 3 weeks had the majority of HMGB1 protein retained within the nucleus (Figures 4B–4D). Strikingly, PS19-E4 mice with 3-week EP + GA treatment also had drastically reduced levels of HMGB1 protein within their hippocampal ISF compared to untreated PS19-E4 mice (Figures 4E and 4F). These data indicate that EP + GA can effectively inhibit the nucleocytoplasmic translocation of HMGB1 while also strongly blocking its cellular release.

We next examined whether EP + GA have effects on other inflammatory cytokines within the ISF. We analyzed the hippocampal ISF from untreated and EP + GA-treated PS19-E4 mice with a multiplex cytokine assay to probe for other cytokines after treatment. This analysis revealed that there were no significant changes in the levels of 19 other inflammatory cytokines in PS19-E4 mice after EP + GA treatment, including disease-relevant cytokines such as TNF-α, IL-1β, and IFN-γ (Figures S6A and S6B). For one of the cytokines, IL-12p70, we noticed a trend toward decreased levels (Figure S6B). Still, there was no correlation between the levels of IL-12p70 in ISF and microgliosis or astrogliosis in untreated and treated PS19-E4 mice (Figures S6C–S6F). This multiplex analysis showed that the small-molecule inhibitors of HMGB1, EP and GA, are potent suppressors of HMGB1 translocation and release in PS19-E4 mice without significantly affecting the levels of other measured inflammatory cytokines in the ISF.

Immunohistochemical analysis of brain tissues from mice utilized for the ISF collection revealed that after the short-term treatment with HMGB1 inhibitors, the treated PS19-E4 mice did not exhibit significant differences in the coverage areas of microglia or astrocytes in the hippocampus compared with untreated PS19-E4 mice (Figures 4G–4J). Intriguingly, we did observe a significant decrease in the coverage areas of activated microglia and astrocytes in treated versus untreated PS19-E4 mice (Figures 4K–4N). This indicates that short-term treatment with HMGB1 inhibitors has a more immediate effect on reducing glial activation states in 9-month-old PS19-E4 mice when gliosis has already developed.

### Long-term treatment with HMGB1 inhibitors ameliorates APOE4-driven gliosis

**Figure 5.**
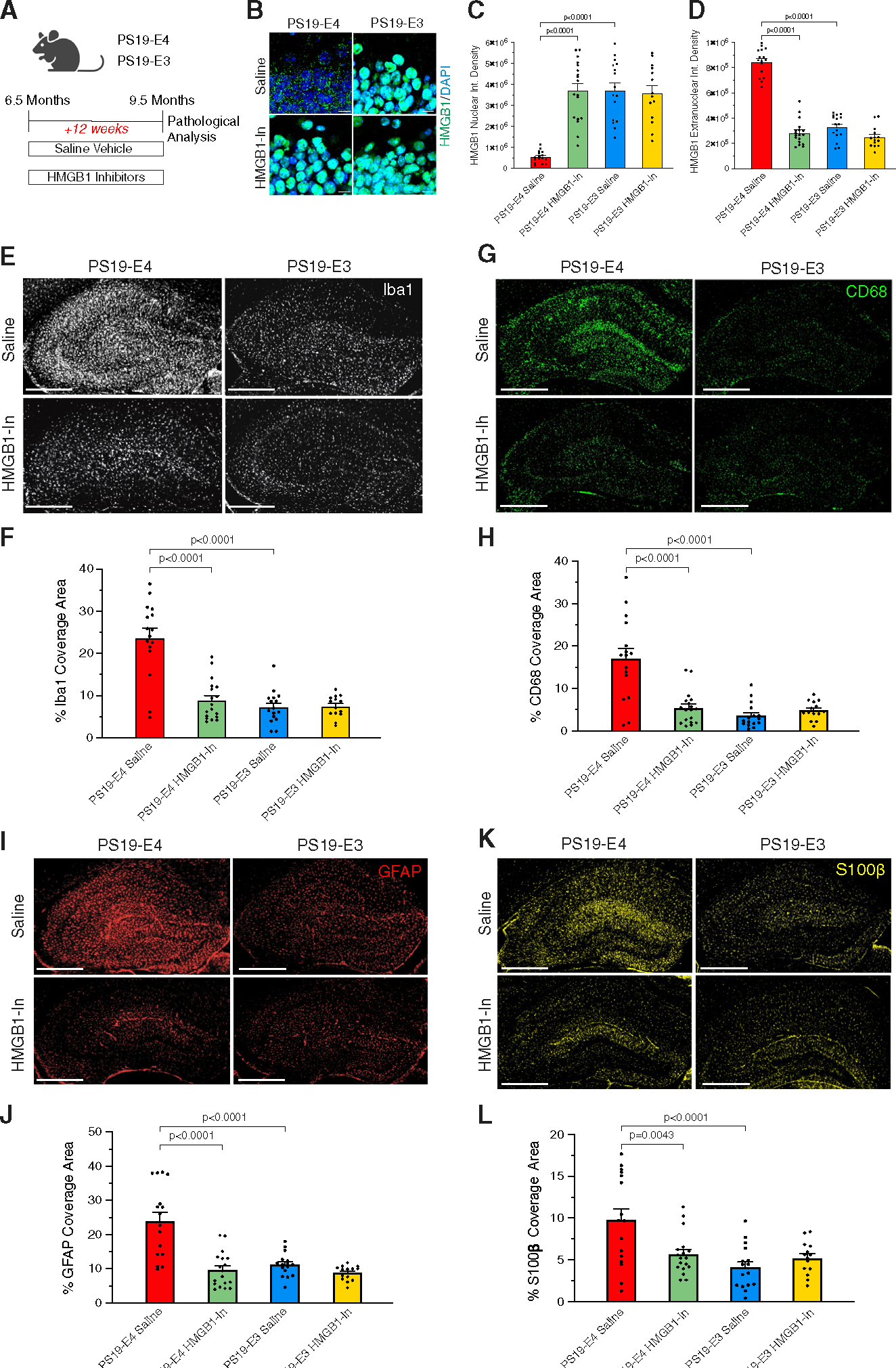
Long-term treatment of PS19-E4 mice with HMGB1 inhibitors blocks the nucleocytoplasmic translocation of HMGB1 in hippocampal neurons and reduces hippocampal gliosis (A) Experimental design of the HMGB1 inhibitor study, with EP + GA treatment at three doses per week. (B) Representative high-magnification images of immunostaining with anti-HMGB1 and DAPI in the dentate gyrus of hippocampus (scale bar, 10 μm). (C and D) Quantification of the nuclear integrated density (C) and extranuclear integrated density (D) of HMGB1 immunostaining in hippocampal neurons. (E) Representative images of microglia immune-staining with anti-Iba1 in the hippocampus (scale bar, 500 μm). (F) Quantification of the percentage of Iba1 coverage area in the hippocampus. (G) Representative images of activated microglia immune-staining with anti-CD68 in the hippocampus (scale bar, 500 μm). (H) Quantification of the percentage of CD68 coverage area in the hippocampus. (I) Representative images of astrocyte immune-staining with anti-GFAP in the hippocampus (scale bar, 500 μm). (J) Quantification of the percentage of GFAP coverage area in the hippocampus. (K) Representative images of activated astrocyte immunostaining with anti-S100β in the hippocampus (scale bar, 500 μm). (L) Quantification of the percentage of S100β coverage area in the hippocampus. For all representative images and quantified data, mice were 9.5 months of age and belonged to either the PS19-E4 or the PS19-E3 group treated with saline or HMGB1 inhibitors. Quantified data in (C), (D), (F), (H), (J), and (L) (PS19-E4 saline, n = 16; PS19-E4 HMGB1-In, n = 18; PS19-E3 saline, n = 16, PS19-E3 HMGB1-In, n = 14) are represented as the mean ± SEM, one-way ANOVA with Tukey’s *post hoc* multiple comparisons test. HMGB1-In, HMGB1 inhibitors.

We then examined the longer-term effects of HMGB1 inhibitors on the development of pathologies in PS19-E mice by administering a mixed solution of EP (80 mg/kg) and GA (20 mg/kg) or saline vehicle to PS19-E4 and PS19-E3 mice at three doses per week for 12 weeks via intraperitoneal injections (Figure 5A). Treatment began when the mice were 6.5 months of age, at about the onset of adverse pathology,^50^ and completed when the mice were 9.5 months of age, which is when severe neurodegeneration and pathological changes are expected to develop, as demonstrated in this and other studies.^23,50^ All mice were monitored for weight changes during the treatment period and no significant changes were observed.

Immunohistochemical analysis of HMGB1 in hippocampal neurons demonstrated that 12-week treatment with the HMGB1 inhibitors significantly reduced the nucleocytoplasmic translocation of HMGB1 in inhibitor-treated PS19-E4 mice relative to the saline control (Figures 5B–5D). In PS19-E3 mice, there was no significant difference in HMGB1 translocation between saline- and inhibitor-treated mice (Figures 5B–5D). Thus, long-term EP + GA treatment can also effectively block APOE4-promoted HMGB1 translocation in hippocampal neurons.

Next, we evaluated the effects of long-term HMGB1 inhibitor treatment on APOE4-driven gliosis. Saline-treated PS19-E4 mice displayed considerable hippocampal microgliosis, whereas long-term HMGB1 inhibitor-treated PS19-E4 mice displayed a significant decrease in microgliosis (Figures 5E–5H). Furthermore, saline-treated PS19-E4 mice also had hippocampal astrogliosis, which was significantly reduced following long-term treatment with HMGB1 inhibitors (Figures 5I–5L). PS19-E3 mice did not display significant differences in microgliosis or astrogliosis whether treated with saline or HMGB1 inhibitors (Figures 5E–5L). These findings demonstrate that long-term treatment with HMGB1 inhibitors ameliorated APOE4-driven gliosis to the levels seen in PS19-E3 mice.

### Long-term treatment with HMGB1 inhibitors ameliorates APOE4-driven Tau pathology, neurodegeneration, and myelin deficits

**Figure 6.**
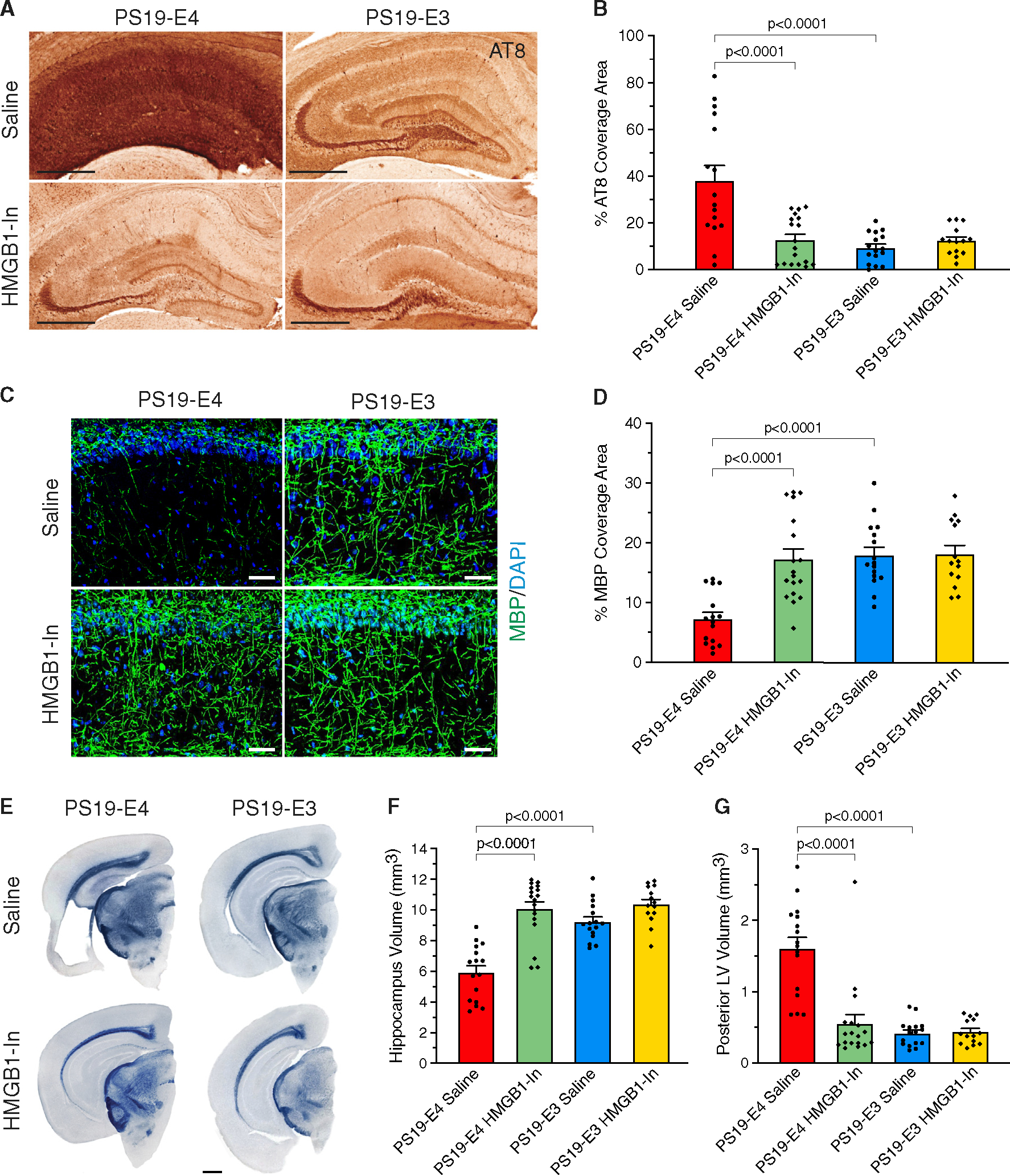
Long-term treatment of PS19-E4 mice with HMGB1 inhibitors drastically reduces Tau pathology, myelin deficits, and hippocampal degeneration (A) Representative images of p-Tau immunostaining with AT8 monoclonal antibody in the hippocampus (scale bar, 500 μm). (B) Quantification of the percentage of p-Tau (AT8^+^) coverage area in the hippocampus. (C) Representative images of myelin sheath staining with anti-MBP and DAPI in the stratum radiatum of CA1 in the hippocampus (scale bar, 100 μm). (D) Quantification of the percentage of MBP coverage area in the stratum radiatum of CA1. (E) Representative images of the ventral hippocampus after staining with Sudan black (scale bar, 1 μm). (F and G) Quantification of hippocampal volume (F) and posterior lateral ventricle volume (G). For all representative images and quantified data, mice were 9.5 months of age and belonged to either the PS19-E4 or the PS19-E3 group treated with saline or HMGB1 inhibitors. Quantified data in (B), (D), (F), and (G) (PS19-E4 saline, n = 16; PS19-E4 HMGB1-In, n = 18; PS19-E3 saline, n = 16, PS19-E3 HMGB1-In, n = 14) are represented as the mean ± SEM, one-way ANOVA with Tukey’s *post hoc* multiple comparisons test. HMGB1-In, HMGB1 inhibitors.

We then analyzed whether the long-term inhibitor treatment can also protect against the development of other AD-relevant pathologies. Saline-treated PS19-E4 mice exhibited substantial Tau pathology throughout the hippocampus, whereas inhibitor-treated PS19-E4 mice had a drastic reduction in Tau pathology (Figures 6A and 6B). There were no discernable differences in Tau pathology between saline- and inhibitor-treated PS19-E3 mice (Figures 6A and 6B). Furthermore, we determined the effectiveness of HMGB1 inhibitors in combating hippocampal myelin deficits and observed that saline-treated PS19-E4 mice displayed severe myelin loss in the stratum radiatum of CA1, while HMGB1 inhibitor-treated PS19-E4 mice displayed a rescue of the deficit with a high coverage area of MBP (Figures 6C and 6D). There were no discernable differences in hippocampal myelin coverage area between saline- and inhibitor-treated PS19-E3 mice (Figures 6C and 6D).

We also determined the effectiveness of long-term treatment with HMGB1 inhibitors at preventing APOE4-driven neurodegeneration. Saline-treated PS19-E4 mice displayed considerable neurodegeneration, whereas HMGB1 inhibitor-treated PS19-E4 mice exhibited a rescue of neurodegeneration as demonstrated by a significant increase in hippocampal volumes and a significant decrease in posterior lateral ventricle volumes (Figures 6E–6G). There were no obvious differences in neurodegeneration between saline- and HMGB1 inhibitor-treated PS19-E3 mice (Figures 6E–6G).

### Long-term treatment with HMGB1 inhibitors diminishes disease-associated microglial subpopulations and enriches disease-protective microglial subpopulations

**Figure 7.**
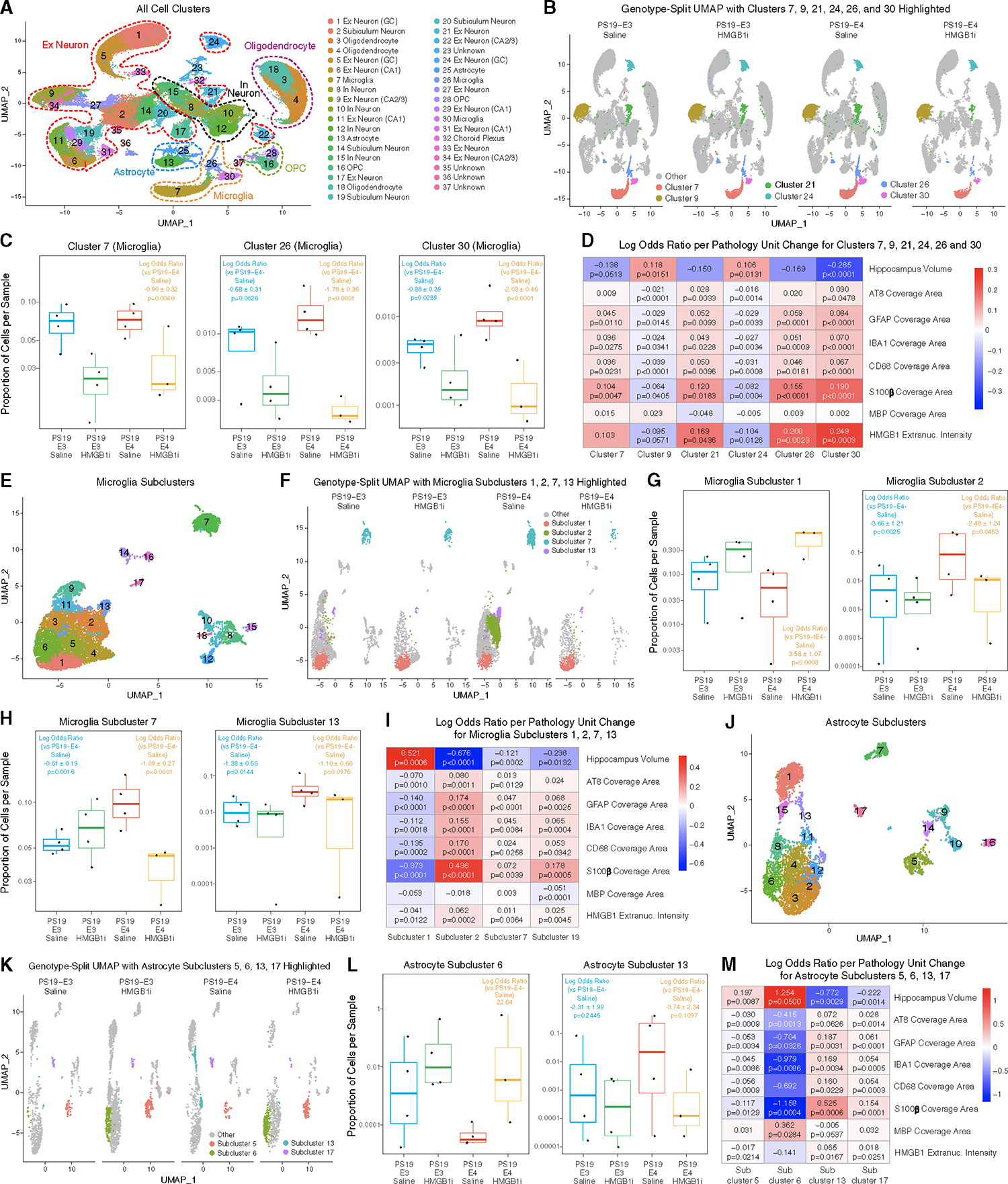
Long-term treatment of PS19-E4 mice with HMGB1 inhibitors diminishes disease-associated and enriches disease-protective subpopulations of microglia and astrocytes (A) UMAP plot of all 37 distinct cell clusters in the isolated hippocampi of 9.5-month-old PS19-E4 mice treated with saline (n = 4) or HMGB1 inhibitors (n = 3) and PS19-E3 mice treated with saline (n = 4) or HMGB1 inhibitors (n = 4). (B) UMAP plot highlighting cells in hippocampal cell clusters 7, 26, and 30 (microglia) and clusters 9, 21, and 24 (excitatory neurons). (C) Boxplot of the proportion of cells from each sample in microglia clusters 7, 26, and 30. (D) Heatmap plot of the log odds ratio per unit change in each pathological parameter for hippocampal cell clusters 7, 9, 21, 24, 26, and 30. (E) UMAP plot of 18 microglial subclusters after subclustering hippocampal cell clusters 7, 26, and 30. (F) UMAP plot highlighting cells in microglial subclusters 1, 2, 7, and 13. (G) Boxplot of the proportion of cells from each sample in microglia subclusters 1 and 2. (H) Boxplot of the proportion of cells from each sample in microglia subclusters 7 and 13. (I) Heatmap plot of the log odds ratio per unit change in each pathological parameter for microglial subclusters 1, 2, 13, and 17. (J) UMAP plot of 17 astrocyte subclusters after subclustering hippocampal cell clusters 13 and 25. (K) UMAP plot highlighting cells in astrocyte subclusters 5, 6, 13, and 17. (L) Boxplot of the proportion of cells from each sample in astrocyte subclusters 6 and 13. There are no cells from PS19-E4 mice in astrocyte subcluster 6, so statistical significance and standard error are not reported. (M) Heatmap plot of the log odds ratio per unit change in each pathological parameter for astrocyte subclusters 5, 6, 13, and 17. For (C), (G), (H), and (L), the log odds ratios are presented as the mean ± SEM. For heatmap plots in (D), (I), and (M), negative associations are shown in blue and positive associations are shown in red. The p values in (C), (G), (H), and (L) are from fits to a GLMM_AM model, and the p values in (D) are unadjusted from fits to a GLMM_histopathology model (see Tables S3, S5, and S7 and STAR Methods for details). All error bars represent the standard error. Ex Neuron, excitatory neuron; In Neuron, inhibitory neuron; OPC, oligodendrocyte progenitor cell. For details, also see Tables S2–S7.

To further analyze the effects of HMGB1 inhibitor treatment in APOE-tauopathy mice, we performed single-nucleus RNA sequencing (snRNA-seq) on hippocampi isolated from 9.5-month-old PS19-E4 and PS19-E3 mice that underwent a 12-week treatment with the HMGB1 inhibitors or saline (Figure S7A). After normalization and filtering for quality control, the snRNA-seq dataset contained 154,803 nuclei covering 26,753 genes (Figures S7B–S7G). snRNA-seq analysis identified 37 distinct cell clusters after clustering by the Louvain algorithm^74^ and visualizing by uniform manifold approximation and projection (UMAP) (Figure 7A). Based on marker gene expression, these 37 cell clusters were assigned to 14 excitatory (Ex) neuron clusters, 4 inhibitory (In) neuron clusters, 4 subiculum neuron clusters, 3 oligodendrocyte clusters, 2 oligodendrocyte progenitor cell (OPC) clusters, 3 microglia clusters, 2 astrocyte clusters, 1 choroid plexus cluster, and 4 unknown clusters (Figures 7A, S7B, and S7C; Table S2). We observed high APOE expression in astrocytes in all mouse groups, regardless of HMGB1 inhibitor treatment (Figure S7H). Microglia and some neurons also expressed APOE, as previously reported.^29,75^ APOE genotypes and HMGB1 inhibitor treatment did not affect the general UMAP profiles (Figures S7I and S7J).

Utilizing log odds ratio estimates from a generalized linear mixed-effects model to assess associations with animal models (GLMM_AM), we identified cell clusters that had different proportions in HMGB1 inhibitor-treated PS19-E4 or PS19-E3 or saline-treated PS19-E3 mice versus saline-treated PS19-E4 mice. Strikingly, all three microglial clusters, 7, 26, and 30, had significantly lower odds of having cells from HMGB1 inhibitor-treated PS19-E4 mice than saline-treated PS19-E4 mice (Figures 7B and 7C; Table S2). Microglial clusters 26 and 30 also had significantly lower odds of having cells from saline-treated PS19-E3 mice than PS19-E4 mice (Figure 7C; Table S2). Compared with saline-treated PS19-E4 mice, the HMGB1 inhibitor-treated PS19-E4 mice had significantly higher odds of having cells in excitatory neuron clusters 9 and 24 and significantly lower odds of having cells in excitatory neuron cluster 21 (Figure S7K; Table S2). Interestingly, excitatory neuron cluster 21 had reduced APOE4 expression and increased SIRT1 expression in HMGB1 inhibitor-treated versus saline-treated PS19-E4 mice (Figure S7L).

The mice utilized for snRNA-seq analysis had also undergone extensive pathological characterization (Figures 5 and 6), as the left brain hemisphere was utilized for RNA-sequencing analysis and the right brain hemisphere was used for pathological analysis. As such, we utilized log odds ratio estimates from another generalized linear mixed-effects model to assess associations with histopathology (GLMM_histopathology) to evaluate the relationships between the transcriptomic data for each cell cluster and pathological measurements in this cohort of mice. This analysis revealed that the proportion of cells in microglial clusters 7 and 30 exhibited significant negative associations with hippocampal volume and positive associations with the coverage areas of microglia and astrocytes (Figure 7D; Table S3). Interestingly, microglial cluster 30 also had significant positive associations with Tau pathology, and microglial clusters 26 and 30 also had significant positive association with the relative amounts of extranuclear HMGB1 (Figure 7D; Table S3).

Furthermore, neuronal clusters 9 and 24 had significant positive associations with hippocampal volume and negative associations with the coverage areas of Tau pathology, microgliosis, and astrogliosis and the relative amounts of extranuclear HMGB1 (Figure 7D; Table S3), while neuronal cluster 21 had positive associations with Tau pathology, gliosis, and extranuclear HMGB1 (Figure 7D; Table S3). These data suggest that microglial clusters 7, 26, and 30 and neuronal cluster 21 are disease-associated glial and neuronal cell clusters, whereas neuronal clusters 9 and 24 are disease-protective neuronal cell clusters. These data also show clearly that treatment of PS19-E4 mice with HMGB1 inhibitors diminishes the presence of disease-associated microglial and neuronal cell clusters while enriching for disease-protective neuron clusters.

To further define the effects of HMGB1 inhibitor treatment on different subsets of microglia, we subclustered microglia clusters 7, 26, and 30 (see Figure 7A) into 18 microglial subpopulations (Figure 7E). Logs odd ratio estimates from a GLMM_AM revealed that microglial subcluster 1 had significantly higher odds and subclusters 2, 7, and 13 had significantly lower odds of having cells from HMGB1 inhibitor-treated than from saline-treated PS19-E4 mice (Figures 7F–7H; Table S4). Microglial subclusters 2, 7, and 13 also had significantly lower odds of having cells from saline-treated PS19-E3 mice than from saline-treated PS19-E4 mice (Figures 7F–7H; Table S4). Interestingly, microglia subclusters 2, 7, and 13 highly expressed APOE (Figure S8A). Intriguingly, comparison of differentially expressed (DE) genes in microglial subcluster 2 between HMGB1 inhibitor-treated and saline-treated PS19-E4 mice (Figures S8B and S8F; Table S4) showed that treatment with HMGB1 inhibitors led to a reversal of the DE gene expression, with the top upregulated genes becoming the top downregulated genes, and vice versa (Figure S8C and S8G; Table S4). We observed a similar reversal of DE gene expression for microglial subcluster 7 (Figures S8D–S8G; Table S4). This indicates that treatment with HMGB1 inhibitors not only diminishes these microglial subclusters, but also leads to a dramatic reversal of DE gene expression in these subclusters.

Log odds ratio estimates from a GLMM_histopathology revealed that microglial subcluster 1 had a significant positive association with hippocampal volume and significant negative associations with the coverage area of AT8^+^ Tau pathology, gliosis, and the relative amounts of extranuclear HMGB1 (Figure 7I; Table S5), suggesting this is a disease-protective microglial subcluster. Conversely, microglial subclusters 2, 7, and 13 had significant negative associations with hippocampus volume and positive associations with Tau pathology, gliosis, and extranuclear HMGB1 (Figure 7I; Table S5), suggesting they are disease-associated microglial (DAM) subclusters. Microglial subcluster 13 also had significant negative associations with the coverage area of MBP (Figure 7I; Table S5). These further suggest that treatment of APOE4-tauopathy mice with HMGB1 inhibitors diminishes the presence of DAM subclusters while enriching for disease-protective microglial subclusters.

Comparison with a recent study that described a subset of DAMs in AD mouse models^76^ with the identified DAMs in our study (subclusters 2, 7, and 13) showed a similar upregulation of gene markers such as Ctsb, APOE, Fth1, and Ctsl (Figure S8F). The expression of many DAM marker genes was reduced in HMGB1 inhibitor-treated versus saline-treated PS19-E4 mice (Figure S8G). On the other hand, we also observed considerable upregulation of a set of genes (Apod, Ttr, Ptgds, Cryab, Plp1, Grik2, Ank2, Malat1) unique to the DAMs identified in this study (Figure S8F), suggesting that these are APOE4-promoted DAM marker genes.

Microglial subclusters 2, 7, and 13 had higher expression levels of Tlr2 (Figure S8H), and subcluster 13 also had higher expression levels of Tlr3 and Tlr7 (Figure S8H), which are major HMGB1 receptors.^31,77^ Accordingly, microglial subclusters 2 and 7 had minor upregulation and microglial subcluster 13 had profound upregulation of Tlr pathway genes (Figure S8I). Microglial subcluster 13 also had clear upregulation of NF-κB pathway genes, which are a downstream target of HMGB1 pathway activation.^31,41^

### Long-term treatment with HMGB1 inhibitors diminishes disease-associated astrocyte subpopulations and enriches disease-protective astrocyte subpopulations

Further subclustering of astrocyte clusters 13 and 25 (see Figure 7A) identified 17 astrocyte subclusters (Figure 7J). Log odds ratio estimates from a GLMM_AM revealed that astrocyte subclusters 5 and 6 had significantly higher odds, while astrocyte subclusters 13 and 17 had significantly lower odds of having cells from HMGB1 inhibitor-treated versus saline-treated PS19-E4 mice (Figures 7K, 7L, and S9B; Table S6). Astrocyte subcluster 13 also had significantly lower odds of having cells from saline-treated PS19-E3 mice than from PS19-E4 mice (Figure 7L; Table S6). Astrocyte subclusters 13 and 17 had relatively high APOE expression (Figure S9A). Strikingly, astrocyte subcluster 6 was entirely absent from saline-treated PS19-E4 mice and this subcluster appeared only after treatment with HMGB1 inhibitors, indicating that the inhibitors induced the formation of this unique astrocyte subcluster in PS19-E4 mice (Figure 7K). Astrocyte subclusters 6 and 13 had unique DE genes relative to other astrocyte subclusters (Figures S9D–S9E; Table S6).

Log odds ratio estimates from a GLMM_histopathology revealed that astrocyte subclusters 5 and 6 have significant positive associations with hippocampal volume and negative associations with Tau pathology, gliosis, and relative amounts of extranuclear HMGB1 (Figure 7M; Table S7), suggesting that they are disease-protective astrocyte subclusters. Meanwhile, astrocyte subclusters 13 and 17 have significant negative associations with hippocampal volume and positive associations with Tau pathology, gliosis, and extranuclear HMGB1 (Figure 7M; Table S7), suggesting that they are disease-associated astrocyte (DAA) subclusters. Astrocyte subcluster 13 also has significant negative associations with the coverage area of MBP (Figure 7M; Table S7).

Comparison with a recent study that described a subset of DAAs in AD mouse models^78^ showed a similar upregulation of some marker genes in DAAs in the current study (Figure S9E), as observed in the previously reported DAAs, supporting the notion that astrocytes in subclusters 13 and 17 are DAAs. The expression of many of these DAA marker genes was reduced in HMGB1 inhibitor-treated versus PS19-E4 mice (Figure S9F). Taken together, these data strongly indicate that treatment of APOE4-tauopathy mice with HMGB1 inhibitors diminishes the presence of DAA subclusters while enriching for disease-protective astrocyte subclusters.

Astrocyte subcluster 17 had higher expression levels of Tlr2, Tlr4, and Tlr7 (Figure S9G), three major HMGB1 receptors.^31,77^ Accordingly, astrocyte subcluster 17 had clear upregulation of Tlr pathway genes (Figure S9H). Astrocyte subcluster 17 also had clear upregulation of NF-κB pathway genes, which are a downstream target of HMGB1 pathway activation.^31,41,42^

## DISCUSSION

In the present study, we demonstrate in a tauopathy mouse model that HMGB1 plays a central role in the induction and exacerbation of APOE4-driven AD pathologies. Specifically, we show that (1) APOE4 leads to significantly more neuronal nucleocytoplasmic translocation and cellular release of HMGB1 than APOE3; (2) higher amounts of extranuclear HMGB1 in neurons induced by APOE4 correlate with more severe microgliosis and hippocampal degeneration; (3) APOE4-driven HMGB1 cellular release acts as a potent inducer of acute and persistent gliosis; (4) removal of neuronal APOE4 reduces HMGB1 intraneuronal translocation and release; (5) treatment of PS19-E4 mice with small-molecule HMGB1 inhibitors (EP + GA) can effectively block APOE4-induced intraneuronal HMGB1 translocation and release and prevent subsequent induction of gliosis; (6) long-term treatment with HMGB1 inhibitors drastically reduces the extent of APOE4-driven gliosis, Tau pathology, myelin deficits, and neurodegeneration in PS19-E4 mice; and (7) HMGB1 inhibitor treatment diminishes disease-associated and enriches disease-protective subpopulations of glial cells in PS19-E4 mice. Taken together, these findings indicate that HMGB1 plays an essential role in the pathogenic mechanism of APOE4-promoted gliosis and subsequent degeneration and that HMGB1 inhibitors represent a promising therapeutic agent to combat APOE4-driven AD and other tauopathies.

APOE4 has a clear effect of promoting neuroinflammation in tauopathy, as we and others have shown that it promotes microgliosis and astrogliosis in mouse models of tauopathy.^7,22,23^ This connection between APOE4 and inflammation is also present in humans, as analysis of *postmortem* AD patient samples shows a greater extent of gliosis throughout the brain^79^ and higher levels of CD68-activated microglia^51^ in APOE4 than in APOE3 carriers. Although it was previously unclear how APOE4 induces neuroinflammation, we show that APOE4 has a compelling effect of promoting the intraneuronal translocation and release of HMGB1, which is a key pro-neuroinflammatory DAMP in the brain.^36,60^ Interestingly and importantly, pharmacological inhibition of HMGB1 intracellular translocation and release reduces not only gliosis, but also Tau pathology in APOE4 tauopathy mice. Since it has been reported that gliosis contributes critically to Tau pathology,^7^ it is plausible that inhibition of APOE4-promoted HMGB1 release leads to reduced gliosis, which in turn contributes to a reduction of Tau pathology.

In the current study, we also observe that APOE4 promotes HMGB1 translocation in astrocytes, but not in microglia, in APOE4-tauopathy mice, which complements a previous study showing that HMGB1 is released from astrocytes in the presence of Tau oligomers.^47^ However, comparing the ratio of extranuclear:nuclear HMGB1 signal in neurons versus astrocytes shows that APOE4 has a much stronger effect of triggering HMGB1 translocation in neurons than in astrocytes. Interestingly, removal of neuronal APOE4 in PS19-E4/Syn1-Cre mice significantly reduced HMGB1 translocation not only in neurons but also in astrocytes, suggesting that the promoted intra-astrocyte translocation of HMGB1 in PS19-E4 mice might be a secondary effect in response to neuronal APOE4’s detrimental actions. In addition, APOE4-KI mice that lack strong Tau pathology do not exhibit neuronal HMGB1 translocation, further strengthening the connection between Tau pathology and HMGB1 in the context of APOE4, especially neuronal APOE4.

We also find that the relative amount of extranuclear HMGB1 in hippocampal neurons, but not in astrocytes or microglia, correlates with the severity of hippocampal degeneration in the context of APOE4, suggesting a link between neuronal HMGB1 translocation (likely also HMGB1 release) and degeneration. This finding is in accordance with a recent small-cohort study of human patients that reported an interactive effect between APOE4 and HMGB1 on reduced cortical thickness in patients with mild cognitive impairment, although this study utilized systemic levels of HMGB1 in the plasma for their comparisons.^26^ Another study found higher levels of HMGB1 within the CSF of some AD patients relative to control individuals.^46^ Considering these studies and our findings that extranuclear HMGB1 (likely also the released HMGB1) correlates with the extent of microgliosis and the severity of hippocampal degeneration in APOE4-tauopathy mice, it is possible that HMGB1 levels in the CSF of human patients could serve as a biomarker of AD progression in APOE4 patients, which is worthy of further study.

Recently, we showed that neuronal APOE4 is a strong driver of Tau pathology, gliosis, and hippocampal degeneration in this PS19 tauopathy mouse model and that the selective removal of APOE4 from neurons protects against the development of these pathologies.^23^ In this study, we demonstrate that removing neuronal APOE4 significantly reduces the extent of neuronal HMGB1 translocation and release into the hippocampal ISF. Mechanistically, we demonstrate that APOE4 leads to increased levels of acetylated HMGB1 and reduced levels of the deacetylase SIRT1 relative to APOE3. We also show that the neuronal APOE4 removal can reverse these mechanistic effects, leading to reduced levels of acetylated HMGB1 and increased levels of SIRT1. This indicates that neuronal APOE4 plays an important role in promoting HMGB1 translocation in neurons and its release from cells into the ISF. Still, it is possible that removing neuronal APOE4 alters many different pathways, so future studies that determine whether selective knockdown of HMGB1 in neurons leads to similar protection in PS19-E4 mice would be useful to strengthen the connection between APOE4 and HMGB1 in neurons. In addition, while we show that neuronal APOE4 has a strong effect on promoting HMGB1 translocation and release, additional studies are required to determine if the protective effects of neuronal APOE4 removal are due fully to its effect on HMGB1.

Recently, concerns have been raised over using a one-size-fits-all approach to AD therapeutic development, and a paradigm shift has been suggested toward developing therapeutics that target specific genetically driven pathogenic mechanisms.^80–82^ Our study supports the notion that targeting the detrimental effects of APOE4 on HMGB1 intracellular translocation and release could serve as a therapeutic approach toward combating AD pathologies that are strongly driven by APOE4. We demonstrate that treatment with two well-characterized small-molecule inhibitors of HMGB1, EP and GA,^70–72^ prevents intraneuronal HMGB1 translocation and release and leads to a striking reduction in a variety of prominent AD pathologies in APOE4-tauopathy mice. We further exemplify the beneficial effects of HMGB1 inhibitor treatment on APOE4-tauopathy mice utilizing snRNA-seq. Our analysis reveals that treatment of PS19-E4 mice with HMGB1 inhibitors significantly diminished the presence of DAMs and DAAs that were enriched in saline-treated PS19-E4 mice and correlated with the relative amounts of extranuclear HMGB1 as well as the severity of Tau pathology, gliosis, and hippocampal degeneration. Intriguingly, HMGB1 inhibitor-treated PS19-E4 mice also had an enrichment of disease-protective subpopulations of microglia and astrocytes that were diminished in saline-treated PS19-E4 mice. These findings provide evidence that treatment with HMGB1 inhibitors represents a novel and effective approach toward the rebalancing of disease-associated and disease-protective glial cells to combat APOE4-driven pathogenesis of AD.

Our pharmacological study also shows that treatment with HMGB1 inhibitors led to significant reductions of AD-related pathologies only in APOE4-tauopathy mice and not in APOE3-tauopathy mice. On one hand, this may suggest that targeting HMGB1 may provide considerable beneficial effects only to patients with the APOE4 genotype, although this requires more extensive studies in different mouse models to validate. On the other hand, our PS19-E3 mice have significantly lower levels of all these pathologies compared with PS19-E4 mice. The differences in pathology between our APOE4- and APOE3-tauopathy mice are in line with the observations in human AD patients that show those with the APOE4 genotype have considerably more microgliosis^51,79^ and astrogliosis,^83^ Tau pathology,^84–86^ neurodegeneration,^25,87^ and myelin deficits^26^ than AD patients with the APOE3 genotype. Still, it is possible that we do not observe a significant therapeutic effect of HMGB1 inhibitors in APOE3-tauopathy mice because they have relatively low pathology to begin with. Therefore, additional studies using different AD mouse models are required to further validate the observation in APOE3-tauopathy mice.

### Limitations of the study

This study also has limitations to consider. While the PS19 mouse model used in this study is widely utilized as a valuable tauopathy model to study AD *in vivo*, this model does not entirely recapitulate the disease processes that occur in human AD. In particular, this model possesses a more virulent form of Tau that expedites disease progression and the development of pathologies compared with the slower disease progression that occurs in human AD patients. As such, the implications of this study for human disease and treatment require further preclinical and clinical investigations. Furthermore, EP and GA are both classified as GRAS (generally regarded as safe) by the US Food and Drug administration (FDA), are found to be safe in humans at clinically relevant doses, and can easily cross the blood-brain barrier in humans.^88–92^ However, more work needs to be done to determine the correct dosage and proper route of administration of these inhibitors in humans to maintain effectiveness against APOE4-driven HMGB1 intracellular translocation and release in future clinical trials. It is also important to note the potential caveat of non-specific effects of EP + GA treatment, as these small molecules may potentially exert off-target effects. As such, more specific HMGB1 inhibitors and/or HMGB1-specific monoclonal antibodies should be developed for future preclinical and clinical studies. In addition, we initiated long-term treatment with HMGB1 inhibitors when the tauopathy mice were 6.5 months of age, which is before they develop severe gliosis or degeneration phenotypes.^50^ When considering the treatment of human APOE4 AD patients with HMGB1 inhibitors or specific anti-HMGB1 antibodies, it will be important to determine at which stage in the disease the inhibitors will be most effective. It is reasonable to speculate that HMGB1 inhibitors would be most effective prior to the onset of severe pathologies, such as in APOE4 patients with mild cognitive impairment. Still, it is possible that the inhibitors would also help slow disease progression even in AD patients that present with severe pathologies since the inhibitors may reduce gliosis and mitigate further degeneration, although this requires additional investigation.

## STAR★METHODS

### RESOURCE AVAILABILITY

#### Lead contact

Further information and requests for resources or reagents should be directed to and will be fulfilled by the lead contact, Yadong Huang (yadong.huang@gladstone.ucsf.edu).

#### Materials availability

Materials generated during this study are accessible via reasonable request to the corresponding author’s lab.

#### Data and code availability

All data associated with this study and the information of used materials are available in the main text, the Materials and Methods, or the supplemental information section. The snRNA-seq datasets generated during the study are available at GEO (accession number: GSE242153). Data associated with Figures 7, S7, S8, and S9, are also available in the supplemental information. All code generated with custom R and shell scripting for this study are available on GitHub at https://github.com/ADNetworksPPG/YH_NK01_APOE4_HMGB1_paper/ and on Zenodo at https://doi.org/10.5281/zenodo.8309839. The two links are also included in the Key Resources Table. Any additional information required to reanalyze the data reported in this paper is available from the Lead Contact upon request.

### EXPERIMENTAL MODEL AND STUDY PARTICIPANT DETAILS

#### Study design

The first objective of this study was to investigate the role of HMGB1 in the pathogenic mechanism of APOE4- versus APOE3-driven AD pathogenesis. We performed pathological characterization of tauopathy mice expressing human APOE4 or APOE3 using immunohistochemical and biochemical analyses. The second objective of the study was to determine the therapeutic efficacy of HMGB1 inhibitors in combating the development of prominent APOE4-driven AD pathologies. We treated APOE4 and APOE3 tauopathy mice with saline or HMGB1 inhibitors and then analyzed their brain tissues by immunohistochemical analyses. Research methods and results are reported in compliance with the Animal Research: Reporting of *In Vivo* Experiments (ARRIVE) guidelines. Sample sizes were chosen on the basis of previous results to allow for adequate statistical power. Mice were matched for age and gender and were randomized to treatment groups. Researchers were blinded to mouse genotype and treatment to exclude the possibility of bias. For specific experiments, refer to the relevant Materials and Methods section and figure legends for outcome measures, statistical methods, and experimental procedures.

#### Mice

We utilized human APOE4 and APOE3 knock-in mice that were generated as previously described.^49^ These APOE knock-in mice contain a LoxP-floxed human *APOE* gene for use in some of the experiments in this and other studies. The APOE knock-in mice were further crossbred with Tau-P301S (PS19) transgenic mice [B6;C3-Tg(Prnp-MAPT*P301S)PS19Vle/J] (The Jackson Laboratory#:008169) expressing human P301S 1N4R Tau driven by the PrP promoter^50^ to generate PS19-E4 and PS19-E3. We also crossbred human LoxP-floxed APOE4 knock-in mice with Synapsin 1-Cre (Syn1-Cre) transgenic mice^69^ [B6.Cg-Tg(Syn1-Cre)671Jxm/J] (The Jackson Laboratory #003966)^97^ and Tau-P301S (PS19) transgenic mice [B6;C3-Tg(Prnp-MAPT*P301S)PS19Vle/J] (The Jackson Laboratory #008169) to generate PS19-E4/Syn1-Cre mice. For generation of the PS19-E4/Syn1-Cre line, only female Syn1-Cre mice were used for breeding purposes because germline recombination has been reported to occur in the progeny of male Syn1-Cre mice.^98^ Wildtype (WT) mice [C57BL/6J] were obtained from the Jackson Laboratory. All mice were on a pure C57BL/6 genetic background and were housed in a pathogen-free barrier facility on a 12 h light cycle at 19–23°C and 30–70% humidity. Animals were identified by ear punch under brief isoflurane anesthesia and genotyped by polymerase chain reaction (PCR) of a tail clipping. All animals otherwise received no procedures except those reported in this study. All animal experiments were conducted in accordance with the guidelines and regulation of the National Institutes of Health, the University of California, and the Gladstone Institutes under the protocol AN176773.

For brain tissue collections, mice were deeply anesthetized with intraperitoneal injections of avertin (Henry Schein) and transcardially perfused for 1 min with 0.9% saline. Brains were either fixed as whole brains or hemi-brains, depending on the study. Right hemi-brains were drop-fixed for 48 h in 4% paraformaldehyde (16% PFA diluted in MilliQ H_2_O) (Electron Microscopy Sciences), rinsed in 1X PBS (Corning) for 24 h, and cryoprotected in 30% sucrose (Sigma) for 48 h at 4°C. The fixed hemi-brains were cut into 30 μm thick coronal sections on a freeze sliding microtome (Leica) and stored in cryoprotectant solution (30% Ethylene Glycol, 30% Glycerol, 40% 1X PBS) at −20°C. Left hemi-brains were snap frozen on dry ice and stored at −80°C.

#### Human donors

Human brain tissue was obtained from donors in the Religious Orders Study or Rush Memory and Aging Project (ROSMAP).^93,99^ Both of these studies enroll individuals free of dementia who participate in an annual clinical evaluation and organ donation at death. Both of these studies were approved by an Institutional Board of Rush University Medical Center, Chicago, IL. All donors signed informed consent and an Anatomical Gift Act, and a repository consent to allow their data to be shared. Demographic information on donors from human AD cohorts can be found in Table S1 More information about accessing ROSMAP data and biosamples can be found at radc.rush.edu.

### METHOD DETAILS

#### Immunohistochemistry on mouse tissues

For immunofluorescent staining, several sections from each mouse (~300 μm apart) were transferred to a 12-well plate in 1X PBS-T (PBS + 0.1% Tween-20) (Millipore Sigma) and were washed 3×5min in PBS-T to remove cryoprotectant solution. Sections were incubated for 5 min in boiling antigen retrieval buffer (Tris buffer, pH 7.6) (TEKNOVA) and washed 2×5min in PBS-T. Sections were then incubated in blocking solution (5% normal donkey serum (Jackson Labs), 0.2% Triton-X (Millipore Sigma) in 1X PBS) for 1 h at room temperature to prevent non-specific antibody binding. After blocking, sections were washed 1×5min in PBS-T and incubated in Mouse-on-Mouse (M.O.M.) Blocking Buffer (1 drop M.O.M IgG/4mL PBS-T) (Vector Labs) for 1 h at room temperature. After M.O.M. block, sections were incubated in primary antibody at 4°C overnight after being diluted to optimal concentrations: anti-CD68 1:100 (Bio-Rad); anti-GFAP 1:800 (Millipore Sigma); anti-HMGB1 1:100 (Abcam); anti-Iba1 (rbt) 1:200 (Wako); anti-Iba1 (goat) 1:100 (Abcam); anti-MAP2 1:200 (Abcam); anti-MBP 1:500 (Abcam); anti-S100β 1:200 (Abcam); anti-SIRT1 1:100 (Abcam). Following primary antibody incubation, sections were washed 3×5min in PBS-T and then incubated in fluorescence-labeled secondary antibodies (Abcam, Jackson Immuno, 1:1000 in PBS-T) for 1 h at room temperature protected from light after being diluted in PBS-T. Sections were then washed 2×5min in PBS-T and incubated in DAPI (1:50,000 in PBS-T) (Thermofisher) for 8 min at room temperature protected from light. Sections were then washed 2×5min in PBS-T, mounted onto microscope slides (Fisher Scientific), coverslipped with ProLong Gold mounting media (Vector Laboratories), and sealed with clear nail polish. Images were taken using an FV3000 confocal laser scanning microscope (Olympus) or Aperio VERSA slide scanning microscope (Leica) at 10X, 20X, 40X, or 60X magnifications depending on the stain. Image analyses of percent coverage area were performed using the open-source Fiji (ImageJ) software after setting a standard threshold value that is applied to all images. Researchers were also blinded to samples to exclude the possibility of bias.

For DAB (3, 3’-diaminobenzidine) staining, several sections from each mouse (~300 μm apart) were transferred to a 12-well plate in 1X PBS-T and then washed 3×5min in PBS-T to remove cryoprotectant solution. Sections were then incubated for 5 min in boiling antigen retrieval buffer (1X PBS, 0.1M sodium citrate, 0.1M citric acid) (Fisher Scientific, Fluka) and washed 2×5min in PBS-T. Next, sections were incubated for 15 min in endogenous peroxidase buffer (1X PBS, 10% methanol (Fisher Scientific), 3% H_2_O_2_ (Sigma) and washed 3×5min in PBS-T. Sections were then incubated in blocking solution (1X PBS-T, 5% normal donkey serum, 1% non-fat dry milk) for 1 h at room temperature. After blocking, sections were washed 2×5min in PBS-T and then incubated in Avidin/Biotin blockage (4 drops of each block) (Vector Laboratories) for 15 min and then washed 2×5min in PBS-T. Sections were incubated in M.O.M. Blocking Buffer (1 drop M.O.M IgG/4mL PBS-T) (Vector Labs) for 1 h at room temperature. Following M.O.M. block, sections were washed 2×5min and incubated in primary antibody at 4°C overnight after being diluted in PBS-T to optimal concentrations (anti-pTau (AT8) 1:100 (Invitrogen). After primary antibody incubation, sections were washed 3×5min in PBS-T and then incubated in biotinylated secondary antibody (1:200; Jackson Immuno) at room temperature for 1 h. Next, sections were washed 3×5min in PBS-T and incubated in ABC buffer (Vector Laboratories) that was prepared 10 min prior to the incubation step. Sections were washed for 2×5min in PBS-T and 1×5min in Tris buffer (pH 7.6). Sections were incubated in DAB buffer (5mL 1X PBS, 2 drops Buffer Stock Solution, 2 drops DAB, 2 drops H_2_O_2_) (Vector Laboratories) for precisely 2 minutes. Staining was halted by washing sections 3×5min in Tris buffer (pH 7.6) and 2×5min in PBS-T. Sections were mounted onto microscope slides and dried at room temperature overnight. Next, mounted sections were submerged into Xylene (Fisher Scientific) 2×5min and coverslipped with DPX mounting media (Sigma-Aldrich). Images were taken using an Aperio VERSA slide scanning microscope (Leica) at 10X magnification.

#### Immunohistochemistry on human tissues

1cm blocks of PFA-fixed prefrontal cortex were obtained from ROSMAP and sectioned at 50um. Several sections from each subject were transferred to a 12-well plate and rinsed with 1X PBS-T (PBS + 0.1% Tween-20) (Millipore Sigma), before being exposed to UV light overnight to reduce autofluorescence. The following day, sections were rinsed three times with 1X PBS-T and incubated for 15 min in boiling antigen retrieval buffer (Tris buffer, pH 8). Sections were then washed three times with 1X PBS-T before incubating in blocking buffer (5% normal donkey serum (Jackson Labs), 0.2% Triton-X (Millipore Sigma) in 1X PBS) for 1 h to prevent non-specific antibody binding. Following block, sections were incubated overnight at 4°C in primary antibody prepared in a solution of 1X PBS-T containing 3% normal donkey serum. The following antibodies were used for labeling human sections: anti-HMGB1 1:100 (Abcam), anti-NeuN 1:200 (ABN90), and anti-GFAP 1:300 (MAB3402). After overnight incubation, sections were again rinsed three times with 1X PBS-T and then incubated in fluorescence-labeled secondary antibodies (Invitrogen, Abcam 1:1000 in PBS-T) for 1 hour in the dark. Following 2 more rinses in 1X PBS-T, sections were incubated in DAPI (1:20,000 in 1X PBS-T) (Thermofisher) for 8 minutes and then washed for a final set of 3 rinses in 1X PBS-T. Sections were mounted onto microscope slides (Fisher Scientific) and coverslipped with ProLong Gold mounting media (Vector Laboratories). After sealing with clear nail polish and allowing slides to dry, sections were imaged on the FV300 confocal laser scanning microscope (Olympus).

#### Volumetric analysis

Serial coronal hippocampal brain sections (7 sections per mouse, 30 μm thick, 300 μm apart) were mounted onto microscope slides (Fisher Scientific) and dried at room temperature for 1 h. The 0.1% Sudan Black solution was prepared by adding the appropriate amount of Sudan Black powder (Sigma) to 70% ethanol (KOPTEC) and mixing the solution using a magnetic stirrer while and protected from light. The solution was then centrifuged at 3,000 RPM for 10 min and the collected supernatant was filtered using a 0.2 μm filter syringe (Thermo Scientific) to remove undissolved dye. Sections were then stained with the 0.1% Sudan Black solution at room temperature for 10 min and washed 3×2min in 70% ethanol and 3×5min in Milli-Q water. Sections were then coverslipped with ProLong Gold mounting media (Invitrogen) and imaged on an Aperio VERSA slide scanning microscope (Leica) at 10X magnification. For hippocampal and posterior lateral ventricle volumetric analyses, the areas of interest were traced in ImageJ using the segmented line tool and the volume was calculated using the formula: volume = (sum of area) * 0.3 μm^22^. The sum of area value was obtained by taking a sum of the quantified area measurements of all 7 brain sections per mouse, roughly between coordinates AP=−1.2 and AP=−3.4.

#### Nuclear-cytoplasmic localization of HMGB1 measurements

Two brain sections (30 μm thick, 300 μm apart) were immunostained with anti-HMGB1 (1:100) and DAPI (1:50,000) as described above. Sections were imaged at 60X magnification with or without 3X Zoom using an FV3000 confocal laser scanning microscope (Olympus). All image processing and quantification was performed on the Fiji (ImageJ) software. Briefly, a 1-pixel median filter was applied to the DAPI channel and an appropriate threshold was set to create a mask of DAPI. The image calculator function was then used to overlay the DAPI mask and HMGB1 channel, which provided the HMGB1 staining that was only localized to the nucleus. After obtaining values for integrated density and particles, the image calculator was used to subtract the DAPI mask from HMGB1, which provided HMGB1 staining that was excluded from the nucleus.

#### Biochemical extraction of brain tissue

The hippocampus was dissected from snap frozen mouse hemi-brains after thawing on ice. For biochemical extraction of tau, the hippocampal tissue was weighed and homogenized using a Polytron immersion disperser homogenizer (Kinematica AG) in ice-cold RAB buffer (G Biosciences) at 10 μL/mg tissue, supplemented by phosphatase inhibitors (Roche) and protease inhibitors (Roche). Samples were then centrifuged using an Optima TLX ultracentrifuge (Beckman Coulter) at 50,000g for 20 min at 4°C and the supernatant was collected as the RAB-soluble fraction. The pellets were resuspended in ice-cold RIPA buffer (Thermo Scientific) at 10 μL/mg tissue and centrifuged at 50,000g for 20 min at 4°C. The supernatant was collected as the RIPA-soluble fraction and the pellet was stored at −80°C for further use. All fractions were stored at −80°C until further analyses. For separating hippocampal tissue into nuclear and cytoplasmic fractions, we utilized a nuclear protein extraction kit (Thermofisher) on isolated hippocampi according to the manufacturer protocol.

#### Western blot analysis

Biochemically extracted mouse hippocampal tissue lysates were loaded onto 12% Bis-Tris SDS-PAGE gels (Invitrogen) and separated by gel electrophoresis at 160V using MOPS buffer. The separated proteins were transferred onto nitrocellulose membranes at 18V for 60 min (Trans-Blot Turbo Transfer System (Bio-rad). Membranes were washed 3×5min in PBS-T and then incubated in Intercept blocking buffer (LI-COR) for 1 h at room temperature to block non-specific binding sites. After blocking, membranes were washed 3×5min in PBS-T and incubated with primary antibody overnight at 4°C (AT8 1:3,000 (Invitrogen), Acetyl-HMGB1 1:1000 (Invitrogen), SIRT1 1:1000 (Abcam), GAPDH 1:5000 (Cell Signaling), TUJ1 1:10,000 (Biolegend)). Membranes were washed 3×5min in PBS-T and incubated in fluorescently-labeled secondary antibody (1:20,000; LI-COR) for 1 h in the dark at room temperature. Resulting bands were detected with the Odyssey CLx infrared imaging system (LI-COR), and the fluorescence intensity of the bands was quantified as a ratio of AT8:TUJ1 signal using the Image Studio software.

#### Measurement of HMGB1 levels with sandwich ELISA

Biochemically extracted mouse hippocampal tissue lysates or collected hippocampal interstitial fluid (ISF) were run according to the provided manufacturer protocols (mouse HMGB1; (Novus Biologicals). Reactions of samples were read on a SpectraMaX M5 spectrophotometer (Molecular Devices) and protein concentrations were determined after interpolating a standard curve and adjusting for dilutions.

#### Multiplex cytokine assay

Collected hippocampal interstitial fluid was run on a V-PLEX Plus mouse cytokine 19-plex assay according to the provided manufacturer protocols (Meso Scale Diagnostics). Reactions of samples were read on an MSD Sector Imager 2400 device and protein concentrations were determined after interpolating a standard curve.

#### Stereotaxic surgery on mice

Mice were anesthetized with an intraperitoneal injection of ketamine (60 mg/kg) and xylazine (30 mg/kg) and maintained on 0.8%–1.0% isofluorane (Henry Schein). Mice were secured in a stereotaxic alignment system model 940 using earbars and a tooth bar (Kopf Instruments). The scalp was prepared by removing hair using Nair and sterilizing with 70% ethanol. The scalp was then cut open using a scalpel and sterilized with 70% ethanol. The cranial sutures were better visualized using 3% hydrogen peroxide. Following identification of Bregma, a unilateral stereotaxic site was drilled with a 0.5 μm microburr (Fine Science Tools) using co-ordinates X = +1.5, Y = −2.1, Z = −2.1, with Z measured from the surface of the brain. Mice were injected with 2μL of the ISF fraction, recombinant HMGB1 (R&D Systems), or saline at a rate of 500 nL/min and allowed to diffuse for 3 min. Following surgery, mice were sutured with nylon monofilament non-absorbable 6–0 sutures (Henry Schein), and administered analgesics buprenorphine (0.0375 mg/kg intraperitoneally), ketophen (5 mg/kg subcutaneously), and saline (500μL intraperitoneally). Mice were monitored on a heating pad until ambulatory and provided Hydrogel for hydration.

#### Microdialysis of mouse hippocampus

Brain interstitial fluid was collected using *in vivo* microdialysis of the hippocampus. Surgical procedures, including pre- and post-operative care, were conducted as described above for stereotaxic surgeries. During the surgery, a unilateral stereotaxic site was drilled with a 1.2mm bone drill bit (BASi) and an AtmosLM guide cannula PEG-4 (Amuza) was stereotaxically implanted above the right hippocampus at coordinates X = +1.5, Y = −2.1, Z = −1.1. The cannula was secured in place using dental cement (GC America), and a temporary PEG-4 AtmosLM dummy probe (Amuza) was inserted and fixed with an AC-5 cap nut screw (Amuza). Two days post-surgery, mice were placed in a microdialysis stand-alone system (BASi) overnight to assimilate, and the following afternoon a 1000kDa AtmosLM collection probe (Amuza) was inserted through the guide cannula into the hippocampus, which extends 1mm father down to Z = −2.1 to target the dentate gyrus. Artificial CSF (Harvard Apparatus) made with 0.15% BSA (Thermo Scientific) was circulated through the system at a rate of 0.5 μL/min using a push-pull method, and ISF was collected in a refrigerated fraction collector (BASi) each hour for roughly 24 hours. To prevent clogging of the tubing, pumps were operated at 10X collection speed for the first two hours before being adjusted to a 0.5 μL/min flow rate. Following completion of ISF collection, mice were euthanized and perfused with 0.9% saline, as described above. The brain was dissected into hemispheres, with the right hemi-brain postfixed for 48 hours in 4% PFA and the left hemi-brain fresh frozen. ISF fractions were frozen at −80°C for further analysis.

#### Short-term treatment with HMGB1 inhibitors

At ~9 months of age, male and female PS19-E4 mice were either left untreated or they received intraperitoneal injections of a mixture of HMGB1 inhibitors: ethyl pyruvate (80mg/kg) (Sigma-Aldrich) and glycyrrhizic acid (20mg/kg) (Sigma-Aldrich) dissolved in 0.9% saline. The mice received three injections per week for 3 weeks, starting at ~9 months of age until they reached 9.7 months of age. All mice were monitored for weight changes, grooming changes, and posture during the experiments and no changes were observed. After 3 weeks of treatment, we collected the hippocampal interstitial fluid of the mice using the methods listed above. Following microdialysis, the animals were perfused and their brain tissue was processed for histopathological analysis, as described above.

#### Long-term treatment with HMGB1 inhibitors

At 6.5 months of age, male and female PS19-E4 and PS19-E3 mice were randomly assigned to the control or treatment group. Mice received intraperitoneal injections with either sterile grade 0.9% saline (Fisher Scientific) or a mixture of HMGB1 inhibitors: ethyl pyruvate (80mg/kg) (Sigma-Aldrich) and glycyrrhizic acid (20mg/kg) (Sigma-Aldrich) dissolved in 0.9% saline. The mice received three injections per week for 12 weeks, starting at 6.5 months of age until they reached 9.5 months of age. All mice were monitored for weight changes, grooming changes, and posture during the experiments and no changes were observed. Following treatment, the animals were perfused and their brain tissue was processed for histopathological analysis, as described above.

#### Single-nuclei preparation for 10x loading

The mouse hippocampus was dissected on ice and placed into a pre-chilled 2 mL Dounce with 1 mL of cold 1X Homogenization Buffer (1X HB) (250 mM Sucrose, 25 mM KCL, 5 mM MgCl_2_, 20 mM Tricine-KOH pH7.8, 1 mM DTT, 0.5 mM Sermidine, 0.15 mM Sermine, 0.3% NP40, 0.2 units/μL RNase inhibitor, ~0.07 tablets/sample Protease inhibitor). Dounce with “A” loose pestle (~10 strokes) and then with “B” tight pestle (~15 strokes). The homogenate was filtered using a 70 μM Flowmi strainer (Eppendorf) and transferred to a pre-chilled 2 mL LoBind tube (Fischer Scientific). Nuclei were pelleted by spinning for 5 min at 4°C at 350 RCF. The supernatant was removed and the nuclei were resuspended in 400 μL 1X HB. Next, 400 μL of 50% Iodixanol solution was added to the nuclei and then slowly layered with 600 μL of 30% Iodixanol solution under the 25% mixture, then layered with 600 μL of 40% Iodixanol solution under the 30% mixture. The nuclei were then spun for 20 min at 4°C at 3,000g in a pre-chilled swinging bucket centrifuge. 200 μL of the nuclei band at the 30%–40% interface was collected and transferred to a fresh tube. Then, 800 μL of 2.5% BSA in PBS plus 0.2 units/μL of RNase inhibitor was added to the nuclei and then were spun for 10 min at 500 RCF at 4C. The nuclei were resuspended with 2% BSA in PBS plus 0.2 units/μL RNase inhibitor to reach ~500 nuclei/μL. The nuclei were then filtered with a 40 μM Flowmi stainer. The nuclei were counted and then ~13,000 nuclei per sample were loaded onto 10x Genomics Next GEM chip G. The snRNA-seq libraries were prepared using the Chromium Next GEM Single Cell 3ʹ Library and Gel Bead kit v3.1 (10x Genomics) according to the manufacturer’s instructions. Libraries were sequenced on an Illumina NovaSeq 6000 sequencer at the UCSF CAT Core.

#### Custom reference genome

PS19 Tau mutant APOE knock-in mouse model^49^ was used for single-nucleus RNA-sequencing (snRNA-seq). The Homo sapiens microtubule associated protein Tau (MAPT) (NCBI Reference Sequence: NM_001123066.4)^100^ and the Homo sapiens APOE are genes of interest for this study. These genes are not expected to be a part of the mouse reference genome, so to quantify the reads aligning to these genes of interest, a custom mouse reference genome was made using the reference mouse genome sequence (GRCm38) from Ensembl (release 98)^101^ and the mouse gene annotation file from GENCODE (release M23),^102^ similar to those used in 10x Genomics Cell Ranger mouse reference package mm10 2020-A. The headers of the Ensembl reference mouse genome sequence fasta file with the chromosome names were modified to match the chromosome names in a fasta file from GENCODE. The annotation GTF file contains entries from non-polyA transcripts that overlap with the protein coding genes. These reads are flagged as multi-mapped and are not counted by the 10x Genomics Cell Ranger v6.1.1 count pipeline.^103^ To avoid this, the GTF file was modified to (1) remove version suffixes from transcript, gene, and exon ids to match the Cell Ranger reference packages, (2) remove non-polyA transcripts. The Homo sapiens MAPT sequence and Homo sapiens APOE sequence were appended as separate chromosomes to the end of the mouse reference genome sequence and the corresponding gene annotations were appended to the filtered mouse reference gene annotation GTF file. The 10x Genomics Cell Ranger v6.1.1 mkref pipeline was used to build the custom reference genome using the modified fasta and GTF file.

#### Pre-processing and clustering of mouse snRNA-seq samples

The snRNA-seq samples included a total of 16 samples with four mice from each of the two genotype groups and treatment conditions (saline-treated PS19-E4, HMGB1 inhibitor-treated PS19-E4, saline-treated PS19-E3, HMGB1 inhibitor-treated PS19-E3). Sequencing was done in two sequencing runs or batches. 12 sample were sequenced in the first run. In the second sequencing run, the same 12 libraries were re-sequenced along with 4 new samples. The demultiplexed fastq files for these samples were aligned to the custom mouse reference genome (see custom reference genome methods for additional descriptions) using the 10x Genomics Cell Ranger v6.1.1 count pipeline,^103^ as described in the Cell Ranger documentation. The include-introns flag for the count pipeline was set to true to count the reads mapping to intronic regions. Individual analysis of the two sequencing runs showed that 6 of the 12 re-sequenced libraries (2 saline-treated PS19-E4, 1 HMGB1 inhibitor-treated PS19-E4, 2 saline-treated PS19-E3, 1 HMGB1 inhibitor-treated PS19-E3) from the same nuclear isolation date in the second sequencing run either had batch effects or had Cell Ranger errors, thus did not pass the quality control assessment. These 6 re-sequenced libraries were not included in further analyses. One HMGB1 inhibitor-treated PS19-E4 sample (one of the new samples from the second sequencing run) had a rounded barcode rank plot indicating a lack of good separation between the cell-associated barcodes and the barcodes associated with empty GEMs, thus did not pass the quality control assessment. This sample was therefore excluded from all further analyses. The demultiplexed fastq files that passed the quality control assessment were aggregated across the two sequencing runs using the 10x Genomics Cell Ranger v6.1.1 count pipeline,^103^ as described in the Cell Ranger documentation. The include-introns flag for the count pipeline was set to true to count the reads mapping to intronic regions.

The filtered count matrices generated by the Cell Ranger count pipeline for 15 samples were processed using the R package for single-nucleus analysis Seurat v4.0.5.^94^ Each sample was pre-processed as a Seurat object and the top 1% of cells per sample with a high number of unique genes, cells with <=200 unique genes, and cells >=0.25% mitochondrial genes were filtered out for each sample. The 15 samples were merged into a single Seurat object and normalization and variance stabilization was performed using sctransform^104^ with the “glmGamPoi” (Bioconductor package version 1.6.0) method^95^ for initial parameter estimation.

Graph-based clustering was performed using the Seurat v4.0.5 functions FindNeighbors and FindClusters. First, the cells were embedded in a k-nearest neighbor (KNN) graph (with k=20) based on the Euclidean distance in the PCA space. The edge weights between two cells were further modified using Jaccard similarity. Next, clustering was performed using the Louvain algorithm implementation in the FindClusters Seurat function. Clustering with 15 PCs and 0.7 resolution resulted in 37 distinct biologically relevant clusters, which was used for further analyses.

#### Cell type assignment

Data visualization using Seurat v4.0.5 in the UMAP space for the 15 samples revealed no batch effects by age, sex, genotype, treatment, date of birth, sequencing run, or nuclear isolation date. The marker genes for each cluster were identified using the FindAllMarkers Seurat function on the SCT assay data. This algorithm uses the Wilcoxon Rank Sum test to iteratively identify differentially expressed genes in a cluster against all the other clusters. Marker genes were filtered to keep only positively expressed genes, detected in at least 25% of the cells in either population and with at least 0.5 log2 fold change. We assigned identities to cell clusters by matching the cell clusters to known cell types with the expression of canonical cell-type-specific genes, the expression of genes identified in publicly available mouse hippocampal single-cell RNA-seq datasets, and the expression of each cluster’s marker genes in a publicly available resource of brain-wide *in situ* hybridization images, as we reported previously.^29^

#### Subclustering of astrocytic and microglial sn-RNA-seq data

The hippocampal cell clusters 13 and 25 were annotated as the astrocyte cells and hippocampal cell clusters 7, 26, and 30 were annotated as the microglial cells. Both these cell types were further sub-clustered. Normalization and variance stabilization was performed using sctransform^104^ with the “glmGamPoi” (Bioconductor package version 1.6.0) method^95^ for initial parameter estimation. Graph-based clustering was performed using the Seurat v4.0.5 functions FindNeighbors and FindClusters. First, the cells were embedded in a k-nearest neighbor (KNN) graph (with k=20) based on the Euclidean distance in the PCA space. The edge weights between two cells were further modified using Jaccard similarity. Next, clustering was performed using the Louvain algorithm implementation in the FindClusters Seurat function. Sub-clustering with 15 PCs and 0.9 resolution resulted in 17 distinct biologically relevant subclusters for astrocytes. Sub-clustering with 15 PCs and 0.9 resolution resulted in 18 distinct biologically relevant microglia subclusters.

#### Differentially expressed (DE) gene analysis

Differentially expressed genes between clusters of interest were identified using FindMarkers Seurat function on the SCT assay data. This algorithm uses the Wilcoxon Rank Sum test to identify differentially expressed genes between two populations. Differentially expressed genes were limited to genes detected in at least 10% of the cells in either population and with at least 0.1 log2 fold change. Volcano plots with log2 fold change and p-value from the differentially expressed gene lists were generated using the EnhancedVolcano R package version 1.12.0.^105^

#### Association between clusters and genotype

A Generalized Linear Mixed-Effects Model to assess association with Animal Models (GLMM_AM) was implemented in the lme4 (v1.1–30) R package^96^ and used to estimate the associations between cluster membership and the mouse model. These models were run separately for each cluster of cells. The GLM model was performed with the family argument set to the binomial probability distribution and the ‘*nAGQ’* parameter set to 10 corresponding to a Laplace approximation for the log-likelihood estimation. Cluster membership of cells by sample was modeled as a response variable by a 2-dimensional vector representing the number of cells from the given sample belonging to and not belonging to the cluster under consideration. The corresponding mouse id from which the cell was derived was the random effect variable and the animal model for this mouse id was included as the fixed variable. The reference animal group was set to saline-treated PS19-E4. The resulting p-values for the estimated log odds ratio across the three animal groups (with respect to the saline-treated PS19-E4 group) and clusters were adjusted for multiple testing using the Benjamini-Hochberg method.^106^ The same method was used for estimating the between cluster association with genotype for astrocyte subclusters and microglia subclusters, with gender being used as a covariate for microglia subcluster analysis. The proportion of cells from a sample in a given cluster were calculated by adding a pseudo count of 0.01 to the number of cells from a sample in a given cluster and dividing by the total number of cells from a sample. These proportion values were plotted as a boxplot grouped by animal group.

#### Association between proportion of cell types and histopathological parameters

A Generalized Linear Mixed-Effects Model to assess association with histopathology (GLMM_histopathology) was implemented in the lme4 (v1.1–27.1) R package^96^ and used to identify cell types whose proportions are significantly associated with changes in histopathology across the samples. These models were performed separately for each combination of the cluster of cells and the four histological parameters: hippocampal volume (mm^3^), the percent of AT8 coverage area, the percent of GFAP coverage area, the percent of Iba1 coverage area, the percent of CD68 coverage area, the percent of S100b coverage area, the percent of MBP coverage area, and the integrated density of extranuclear HMGB1. The GLM model was performed with the family argument set to the binomial probability distribution family and the ‘*nAGQ’* parameter set to 1 corresponding to a Laplace approximation for the log-likelihood estimation. Cluster membership of cells by sample was modeled as a response variable per sample according to the number of cells from the given sample belonging or not to the cluster under consideration. The corresponding mouse model with given saline or HMGB1 treatment from which the cell was derived was included as a random effect and further the mouse id within the given mouse model-treatment combination was modeled as a random effect as well. Note, this represents the hierarchical nature of this data for the GLMM, and the mouse models are first assumed to be sampled from an “universe” of mouse models, this is then followed by sampling mice within each mouse model. The modeling choice of including the mouse model as a random effect as opposed to a fixed effect is meant to increase the degrees of freedom (or maximize the statistical power) to detect the association of interest, particularly in light of the relatively small number of replicates (3–4) per animal model. The histological parameter under consideration was modeled as a fixed effect in this model.

We visualized the log odds ratio estimates (derived from the GLMM fits) in heatmaps using *pheatmap* package 1.0.12 after adjusting the p-values distribution across histopathological parameters across cell types with *Benjamini-Hochberg* multiple testing correction.^106^ We also applied the pipeline to the astrocyte and microglia subtypes and visualized the associations between astrocyte and microglia subtypes, with gender being used as a covariant for microglia analysis, and the eight histopathological parameters.

### QUANTIFICATION AND STATISTICAL ANALYSIS

All plotted data are presented as the mean ± SEM, unless otherwise specified. Data were analyzed using unpaired two-sided t test and ordinary one-way analysis of variance (ANOVA) followed by Tukey’s multiple comparisons test. We utilized two-sided tests and all analyzed data met the assumption for the specific statistical test that was performed. The correlations between two data in the same genotype group were analyzed using simple linear regression and plotted as the mean ± SEM. Probability levels of P < 0.05 were considered statistically significant. The analyses were performed and plots were created with GraphPad Prism version 9.2.0.

## Supplementary Material

1

2

3

4

5

6

7

## Figures and Tables

**KEY RESOURCES TABLE:** 

REAGENT or RESOURCE	SOURCE	IDENTIFIER

Antibodies		

Mouse anti-AT8	Thermo Fisher Scientific	Cat#: MN1020; RRID: AB_223647
Rat anti-CD68	Bio-Rad	Cat#: MCA1957; RRID: AB_322219
Mouse anti-GAPDH	Cell Signaling	Cat#: 97166; RRID: AB_2756824
Mouse anti-GFAP	Millipore	Cat#: MAB3402; RRID: AB_94844
Rabbit anti-HMGB1	Abcam	Cat#: ab18256; RRID: AB_444360
Rabbit anti-Acetyl-HMGB1 (Lys29)	Thermo Fisher Scientific	Cat#: PA5–120416 RRID: AB_2913988
Rabbit anti-Iba1	Wako	Cat#: 019–19741; RRID: AB_839504
Rabbit anti-MBP	Abcam	Cat#: ab40390 RRID: AB_1141521
Goat anti-Iba1	Abcam	Cat#: ab5076 RRID: AB_2224402
Guinea Pig anti-NeuN	Millipore	Cat#: ABN90; RRID: AB_11205592
Mouse anti-SIRT1	Abcam	Cat#: ab110304 RRID: AB_10864359
Rabbit anti-S100β	Abcam	Cat#: ab52642; RRID: AB_882426
Rabbit anti-TUJ1	Biolegend	Cat#: 802001; RRID: AB_2564645
Donkey anti-mouse Biotin-SP	Jackson Immuno Research Labs	Cat#: 715-065-150; RRID: AB_2307438
Donkey anti-mouse Alexa Fluor 488	Abcam	Cat#: ab150105; RRID: AB_2732856
Donkey anti-rabbit Alexa Fluor 488	Abcam	Cat#: ab150073; RRID: AB_2636877
Donkey anti-rat Alexa Fluor 488	Abcam	Cat#: ab150153; RRID: AB_2737355
Donkey anti-mouse Alexa Fluor 594	Abcam	Cat#: ab150108 RRID: AB_2732073
Donkey anti-rabbit Alexa Fluor 594	Abcam	Cat#: ab150076 RRID: AB_2782993
Donkey anti-guinea pig 594	Jackson Immuno Research Labs	Cat#: 706-585-148; RRID: AB_2340474
Donkey anti-mouse 647	Abcam	Cat#: ab150107; RRID: AB_2890037
Donkey anti-rabbit 647	Abcam	Cat#ab150075; RRID: AB_2752244
Donkey anti-guinea pig 647	Jackson Immuno Research Labs	Cat#: 706–605-148; RRID: AB_2340476
Donkey anti-mouse IRDye 800CW	LI-COR	Cat#: 926–32212; RRID: AB_621847
Donkey anti-rabbit IRDye 680RD	LI-COR	Cat#: 926–68073; RRID: AB_10954442

Chemicals, peptides, and recombinant proteins		

Artificial CSF	Harvard Apparatus	Cat#: 59-7316
Biotinylated donkey anti-mouse	Jackson Immuno Research Labs	Cat#: 715-065-150
Benzonase^®^ Nuclease	Sigma-Aldrich	Cat#: E1014
cOmplete Protease Inhibitor Cocktail	Roche	Cat#: 11836145001
DAPI	Thermofischer	Cat#: 62248
DPX mounting medium	Sigma-Aldrich	Cat#: 6522
Ethyl Pyruvate	Sigma-Aldrich	Cat#: 8066170500
Glycyrrhizic Acid	Sigma-Aldrich	Cat#: 50531–50G
Intercept Blocking Buffer (PBS)	LI-COR	Cat#: 927–70001
M.O.M. blocking buffer	Vector Labs	Cat#: MKB-2213–1
NewBlot IR Stripping Buffer	LICOR	Cat#: 928–40028
Normal Donkey Serum	Jackson Immuno Research Labs	Cat#: 17000121
NuPage 12% Bis-Tris gel	Invitrogen	Cat#: NP0343
NuPage MOPS SDS Running Buffer	Novex	Cat#: NP0001
Paraformaldehyde	Electron Microscopy Sciences	Cat#: 15710-S
ProLong Gold mounting medium	Vector Labs	Cat#: P36930
PhosSTOP phosphatase inhibitor	Roche	Cat#: 04–906-845–001
RAB buffer	G Biosciences	Cat#: 786–91
Recombinant Human HMGB1 protein	R&D Systems	Cat#: 1690-HMB-050
RIPA buffer	Thermo Fisher Scientific	Cat#: 89900
Saline (0.9%)	Fisher Scientific	Cat#: Z1376
Sudan Black B	Sigma-Aldrich	Cat#: 199664

Critical commercial assays		

Avidin/Biotin blocking kit	Vector Labs	Cat#: SP-2001
ABC-HRP Kit, Peroxidase	Vector Labs	Cat#: PK-6100
DAB Substrate Kit	Vector Labs	Cat#: SK-4100
Chromium Next GEM Single Cell 3' Library and Gel Bead kit v3.1	10x Genomics	Cat#: 1000128
Mouse HMGB1 ELISA kit	Novus Biologicals	Cat#: NBP2–62767
V-PLEX Plus Mouse Cytokine 19-Plex kit	Meso Scal Diagnostics	Cat#: K15255G
NE-PER nuclear and cytoplasmic extraction reagents	Thermo Fisher Scientific	Cat#: 78835

Deposited data		

Raw snRNA-seq data	This paper	GEO accession number: GSE242153
Processed snRNA-seq data	This paper	Tables S1, S2, S3, S4, S5, S6

Experimental models: Organisms/strains		

Mouse: APOE3-KI: Apoetm2(APOE_i3)Yhg	Yadong Huang	Bien-Lyet. al.^49^, 2012
Mouse: APOE4-KI: Apoetm3(APOE_i4)Yhg	Yadong Huang	Bien-Lyet. al.^49^, 2012
Mouse; PS19-E4 APOE4-KI crossbred to Tau-P301S (PS19 line) (Jax #008169)	This paper	N/A
Mouse: PS19-E3 APOE3-KI crossbred to Tau-P301S (PS19 line) (Jax #008169)	This paper	N/A
Mouse: PS19-E4/Syn1-Cre APOE4-KI crossbred to Tau P301S (PS19 line; Jax #008169) crossbred to Syn1-Cre (Jax 003966)	This paper	N/A
Mouse: Wildtype	The Jackson Laboratory	Cat#: 000664
Human: Brain tissue from ROSMAP cohort	Bennett et al^93^2018	radc.rush.edu

Oligonucleotides		

Software and algorithms		

GraphPad Prism 9 for Mac	GraphPad Software	https://www.graphpad.com/scientific-software/prism/
Fiji v2.3	Schindelin et al, 2012	https://imagej.net/software/fiji/downloads
Image Studio Lite v.5.2.5	LI-COR	https://www.licor.com/bio/image-studio-lite/resources
Seurat v4.0.5	Hao et al^94^, 2021	https://satijalab.org/seurat/artides/install.html
Cellranger v6.1.1	10x Genomics	https://support.10xgenomics.com/single-cell-gene-expression/software/downloads/6.1/
glmGamPoi v1.6.0	Ahlmann-Eltze et al^95^, 2021	https://bioconductor.org/packages/release/bioc/html/glmGamPoi.html
lme4 v.1.1–27.1	Bates etal^96^, 2015	https://cran.r-project.org/web/packages/lme4/index.html
lme4 v.1.1–30	Bates etal^96^, 2015	https://cran.r-project.org/web/packages/lme4/index.html
R v4.1.2	R Core Team	https://www.R-project.org
Codes generated in this study	This paper	https://github.com/ADNetworksPPG/YH_NK01_APOE4_HMGB1_paper/
Codes generated in this study	This paper	https://doi.org/10.5281/zenodo.8309839.

Other		

Aperio VERSA slide scanning microscope	Leica	N/A
AtmosLM Collection Probe (1000 kDa)	Eicom	Cat#:PEP-4–01
Bone drill bit (1.2mm)	BASi	Cat#: MD-1360
Fraction Collector	BASi	Cat#: MD-1201
FV3000 confocal laser scanning microscope	Olympus	N/A
GC FujiCEM 2	GC America	Cat#: 441001
Microdialysis stand-alone system	BASi	Cat#: MD-1409
Nylon monofilament non-abaorbable 6–0 sutures	Henry Schein	Cat#: 5617265
Odyssey CLx Imaging System	LI-COR	N/A
Optima TLX Ultracentrifuge	Beckman Coulter	N/A
PEG-4 AtmosLM dummy probe	Amuza	Cat#: 600.134.00
PEG-4 AtmosLM guide cannula	Amuza	Cat#: 600.133.00
Polytron PT-MR 3100D homogenizer	Kinematica AG	Cat: #PF-768-0025-04-02
Spectrophotometer SpectraMax M5	Molecular Devices	N/A
Stereotaxic alignment system model 940	Kopf Instruments	N/A
Trans-blot turbo transfer system	BIO-RAD	Cat#: 1704150
Sector imager 2400	Meso Scale Diagnostics	N/A

## Highlights

- APOE4 promotes neuronal HMGB1 translocation and release in tauopathy mice
- Neuronal APOE4 removal reduces HMGB1 translocation and release in tauopathy mice
- Treatment with HMGB1 inhibitors ameliorates APOE4-driven gliosis and AD pathologies
- The inhibitor treatment reduces disease-associated neuronal and glial subtypes
