# Supplementary material for: APOE4-promoted gliosis and degeneration in tauopathy are ameliorated by pharmacological inhibition of HMGB1 release
Source: Cell Rep. Author manuscript; Available in PMC 2024 Feb 16. (PMC10873109; doi:10.1016/j.celrep.2023.113252)
Supplement: 1 [file NIHMS1941924-supplement-1.pdf]

Cell Reports, Volume *42*

Supplemental information

APOE4-promoted gliosis and degeneration in tauopathy are ameliorated by pharmacological inhibition of HMGB1 release

Nicole Koutsodendris, Jessica Blumenfeld, Ayushi Agrawal, Michela Traglia, Oscar Yip, Antara Rao, Min Joo Kim, Maxine R. Nelson, Yung-Hua Wang, Brian Grone, Yanxia Hao, Reuben Thomas, Misha Zilberter, Seo Yeon Yoon, Patrick Arriola, and Yadong Huang

Supplemental Information (SI) Table of Contents

Figure S1, in this SI document.

Figure S2, in this SI document.

Figure S3, in this SI document.

Figure S4, in this SI document.

Figure S5, in this SI document.

Figure S6, in this SI document.

Figure S7, in this SI document.

Figure S8, in this SI document.

Figure S9, in this SI document.

Table S1. Demographic information of samples used in Figure 1I–1K, in this SI document.

Table S2. snRNA-seq data related to all cell clusters, including marker genes, log odds ratios of cell clusters across genotypes and treatments, and cell counts per sample per cluster, as an excel spreasheet file.

Table S3. snRNA-seq data related to all cell clusters, including log odd ratio estimates of associations with histopathology for all cell clusters and differentially expressed (DE) pathways for some cell clusters, as an excel spreasheet file.

Table S4. snRNA-seq data related to microglial subclusters, including log odds ratios of subclusters across genotypes and treatments, cell counts per sample per cluster, and differentially expressed (DE) genes and pathways for some microglial subclusters, as an excel spreasheet file.

Table S5. snRNA-seq data of log odd ratio estimates of associations with histopathology for all microglial subclusters, as an excel spreasheet file.

Table S6. snRNA-seq data related to astrocyte subclusters, including log odds ratios of subclusters across genotypes and treatments, cell counts per sample per cluster, and differentially expressed (DE) genes for some microglial subclusters, as an excel spreasheet file.

Table S7. snRNA-seq data of log odd ratio estimates of associations with histopathology for all astrocyte subclusters, as an excel spreasheet file.

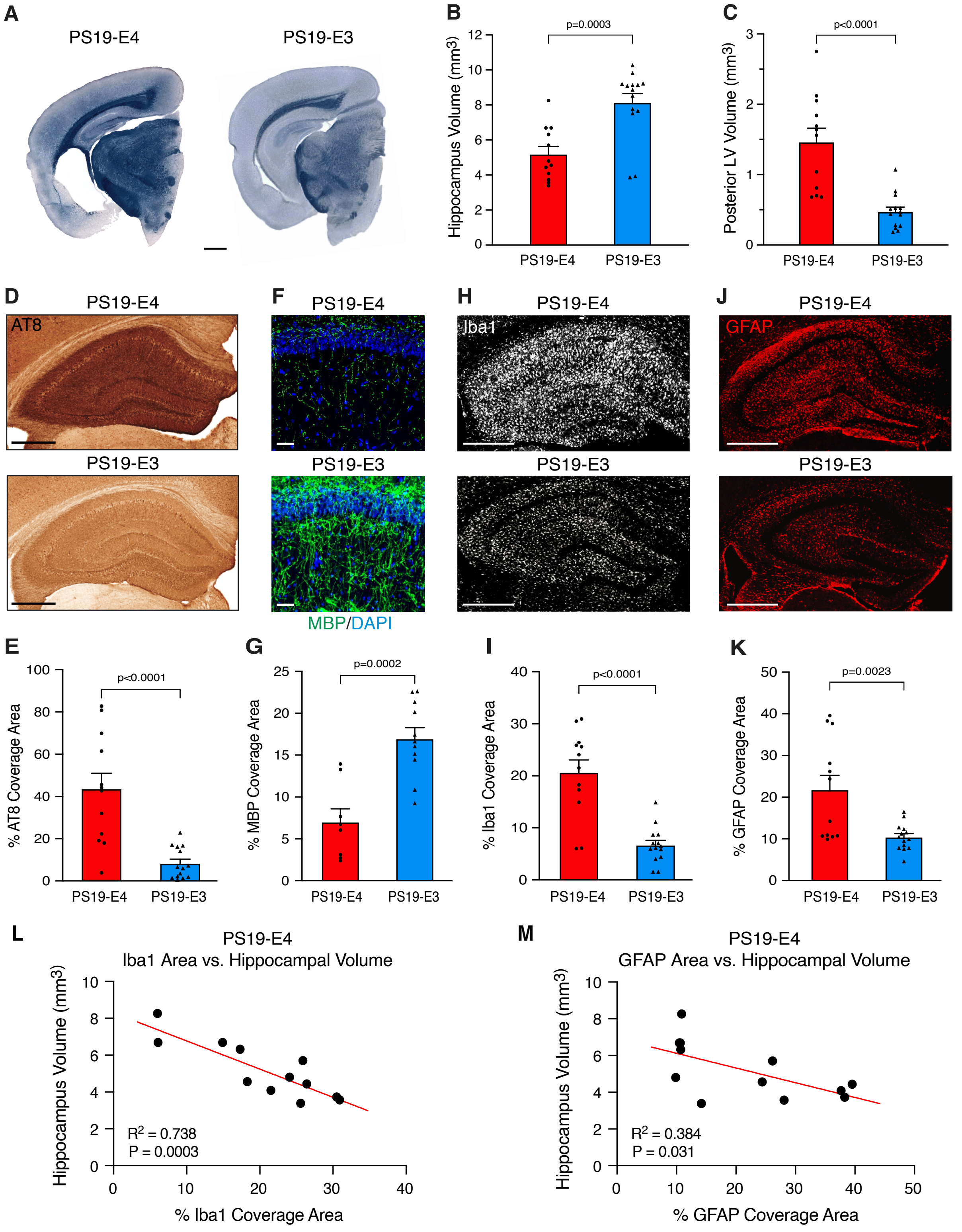

Figure S1. APOE4 increases neurodegeneration, Tau pathology, myelin deficits, and gliosis relative to APOE3 in a mouse model of tauopathy.

(**A**) Representative images of the ventral hippocampus after staining with Sudan Black (scale bar, 1 mm).

(**B and C**) Quantification of hippocampal volume (B) and posterior lateral ventricle volume (C).

(**D**) Representative images of p-Tau staining with AT8 monoclonal antibody in the hippocampus (scale bar, 500 μm).

(**E**) Quantification of the percent p-Tau (AT8$^+$) coverage area in the hippocampus.

(**F**) Representative images of myelin basic protein (MBP) immunostaining with anti-MBP in the stratum radiatum of the hippocampus underneath the pyramidal cell layer of CA1 (scale bar, 100 μm).

(**G**) Quantification of the percent MBP coverage area in the stratum radiatum of the hippocampus.

(**H**) Representative images of microglia immunostaining with anti-Iba1 in the hippocampus (scale bar, 500 μm).

(**I**) Quantification of the percent Iba1 coverage area in the hippocampus.

(**J**) Representative images of astrocyte immunostaining with anti-GFAP in the hippocampus (scale bar, 500 μm).

(**K**) Quantification of the percent GFAP coverage area in the hippocampus.

(**L**) Correlation between the percent Iba1 coverage area and hippocampal volume in PS19-E4 mice.

(**M**) Correlation between the percent GFAP coverage area and hippocampal volume of PS19-E4 mice.

For all representative images and quantified data, mice were 10-months-old and belonged to either the PS19-E4 or PS19-E3 group. Quantified data in B,C,E,I,K (PS19-E4, n=12; PS19-E3, n=14) and G (PS19-E4, n=8, PS19-E3, n=11) are represented as mean±SEM, unpaired two-sided t test. Data in L,M (PS19-E4, n=12) are Pearson's correlation analysis (two-sided).

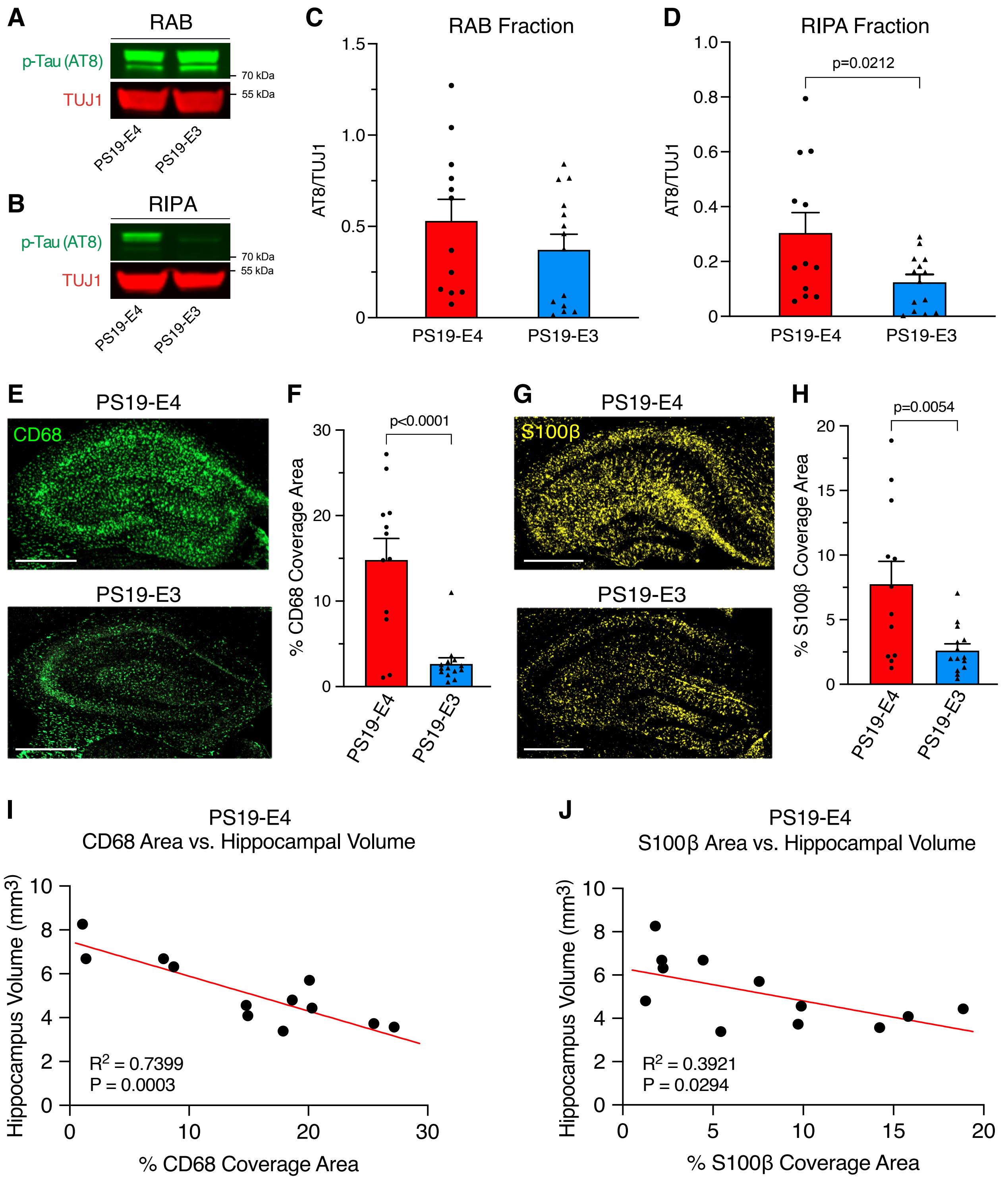

Figure S2. APOE4 increases p-Tau levels as well as the coverage area of activated microglia and activated astrocytes relative to APOE3 in a mouse model of tauopathy.

(**A and B**) Representative images of anti-p-Tau (AT8, green) and anti-TUJ1 (red) western blots in RAB (A) and RIPA (B) fractions of hippocampal tissue lysates.

(**C and D**) Quantification of AT8-positive p-Tau levels relative to TUJ1 measured by western blot analysis in RAB (C) and RIPA (D) fractions of the hippocampal lysates.

(**E**) Representative images of activated microglia immunostaining with anti-CD68 in the hippocampus (scale bar, 500 µm).

(**F**) Quantification of the percent CD68 coverage area in the hippocampus.

(**G**) Representative images of activated astrocyte immunostaining with anti-S100β in the hippocampus (scale bar, 500 µm).

(**H**) Quantification of the percent S100β coverage area in the hippocampus.

(**I**) Correlation between the percent CD68 coverage area and hippocampal volume.

(**J**) Correlation between the percent S100β coverage area and hippocampal volume.

For all representative images and quantified data, mice were 10-months-old and belonged to either the PS19-E4 or PS19-E3 group. Quantified data in C,D (PS19-E4, n=12; PS19-E3, n=14) are represented as mean±SEM, unpaired two-sided t test. Data in I,J (PS19-E4, n=12) are Pearson's correlation analysis (two-sided).

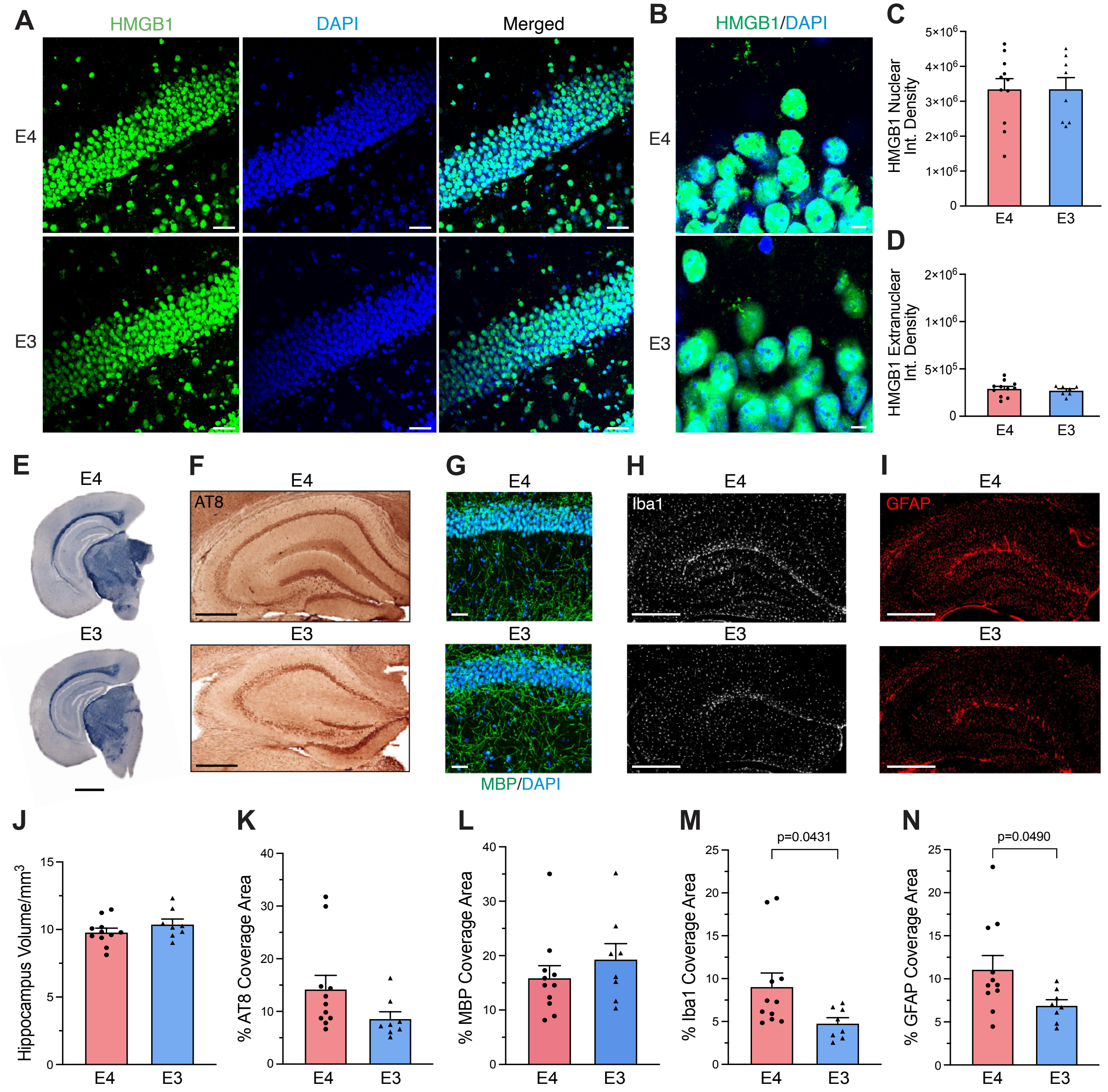

Figure S3. APOE4 does not induce the nucleo-cytoplasmic translocation of HMGB1 in hippocampal neurons in mice without tauopathy, Related to Figure 1.

(**A**) Representative images of immunostaining with anti-HMGB1 and DAPI in the dentate gyrus (scale bar, 40 μm).

(**B**) Representative high magnification images of immunostaining with anti-HMGB1 and DAPI in the dentate gyrus (scale bar, 10 μm).

(**C**) Quantification of nuclear integrated density of HMGB1 immunostaining in hippocampal neurons.

(**D**) Quantification of extranuclear integrated density of HMGB1 immunostaining in hippocampal neurons.

(**E**) Representative images of the ventral hippocampus after staining with Sudan Black (scale bar, 1 mm).

(**F**) Quantification of hippocampal volume

(**G**) Representative images of p-Tau staining with AT8 monoclonal antibody in the hippocampus (scale bar, 500 μm).

(**H**) Quantification of the percent p-Tau ($AT8^+$) coverage area in the hippocampus.

(**I**) Representative images of myelin basic protein (MBP) immunostaining with anti-MBP in the stratum radiatum of the hippocampus underneath the pyramidal cell layer of CA1 (scale bar, 100 μm).

(**J**) Quantification of the percent MBP coverage area in the stratum radiatum of the hippocampus.

(**K**) Representative images of microglia immunostaining with anti-Iba1 in the hippocampus (scale bar, 500 μm).

(**L**) Quantification of the percent Iba1 coverage area in the hippocampus.

(**M**) Representative images of astrocyte immunostaining with anti-GFAP in the hippocampus (scale bar, 500 μm).

(**N**) Quantification of the percent GFAP coverage area in the hippocampus.

For all representative images and quantified data, mice were 10-months-old and belonged to either the APOE4-KI (E4) or APOE3-KI (E3) group, which do not carry P301S mutant Tau. Quantified data in C,D,E,F,H,J,L,N,P (E4, n=11; E3, n=8) are represented as mean±SEM, unpaired two-sided t test.

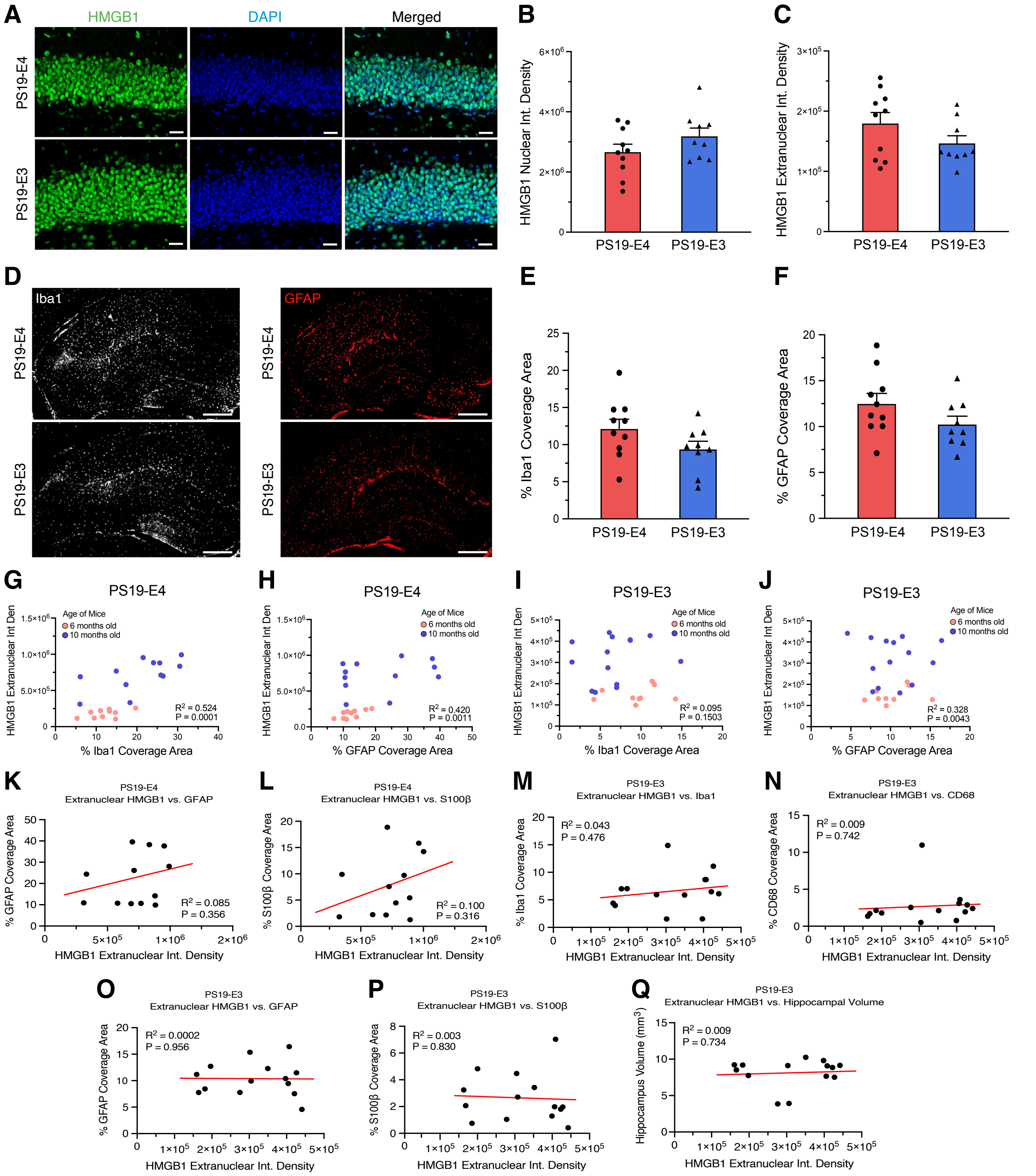

Figure S4. No difference in HMGB1 intracellular translocation in young PS19-E4 and PS19-E3 mice and the relative amounts of extranuclear HMGB1 does not correlate with the coverage area of astrocytes in old PS19-E4 mice, Related to Figure 1.

(**A**) Representative images of immunostaining with anti-HMGB1 and DAPI in the dentate gyrus of 6-month-old PS19-E mice (scale bar, 40 µm).

(**B**) Quantification of nuclear integrated density of HMGB1 immunostaining in hippocampal neurons of 6-month-old PS19-E mice.

(**C**) Quantification of extranuclear integrated density of HMGB1 immunostaining in hippocampal neurons of 6-month-old PS19-E mice.

(**D**) Representative images of microglia immunostaining with anti-Iba1 and astrocyte immunostaining with anti-GFAP in the hippocampus of 6-month-old PS19-E mice (scale bar, 500 μm).

(**E**) Quantification of the percent Iba1 coverage area in the hippocampus of 6-month-old PS19-E mice.

(**F**) Quantification of the percent GFAP coverage area in the hippocampus of 6-month-old PS19-E mice.

(**G**) Multiple variables correlation between extranuclear HMGB1 integrated density, percent Iba1 coverage area, and age in PS19-E4 mice.

(**H**) Multiple variables correlation between extranuclear HMGB1 integrated density, percent GFAP coverage area, and age in PS19-E4 mice.

(**I**) Multiple variables correlation between extranuclear HMGB1 integrated density, percent Iba1 coverage area, and age in PS19-E3 mice.

(**J**) Multiple variables correlation between extranuclear HMGB1 integrated density, percent GFAP coverage area, and age in PS19-E3 mice.

(**K and L**) Correlations between the relative amounts of HMGB1 extranuclear density and percent of GFAP coverage area (K), percent of S100β coverage area (L) in 10-month-old PS19-E4 mice.

(**M–Q**) Correlations between the relative amounts of HMGB1 extranuclear density and percent of Iba1 coverage area (M), percent of CD68 coverage area (N), percent of GFAP coverage area (O), and percent of S100β coverage area (P), hippocampal volume (Q) in 10-month-old PS19-E3 mice.

Quantified data in B,C,E,F (E4, n=10; E3, n=9) are represented as mean±SEM, unpaired two-sided t test. Data in G,H,I,J, (6-month-old PS19-E4, n=10; 10-month-old PS19-E4, n=12; 6-month-old PS19-E3, n=9; 10-month-old PS19-E3, n=14) are analyzed by multiple linear regression analysis. Data in K,L (PS19-E4, n=12) and M,N,O,P,Q (PS19-E3, n=14) are Pearson's correlation analysis (two-sided).

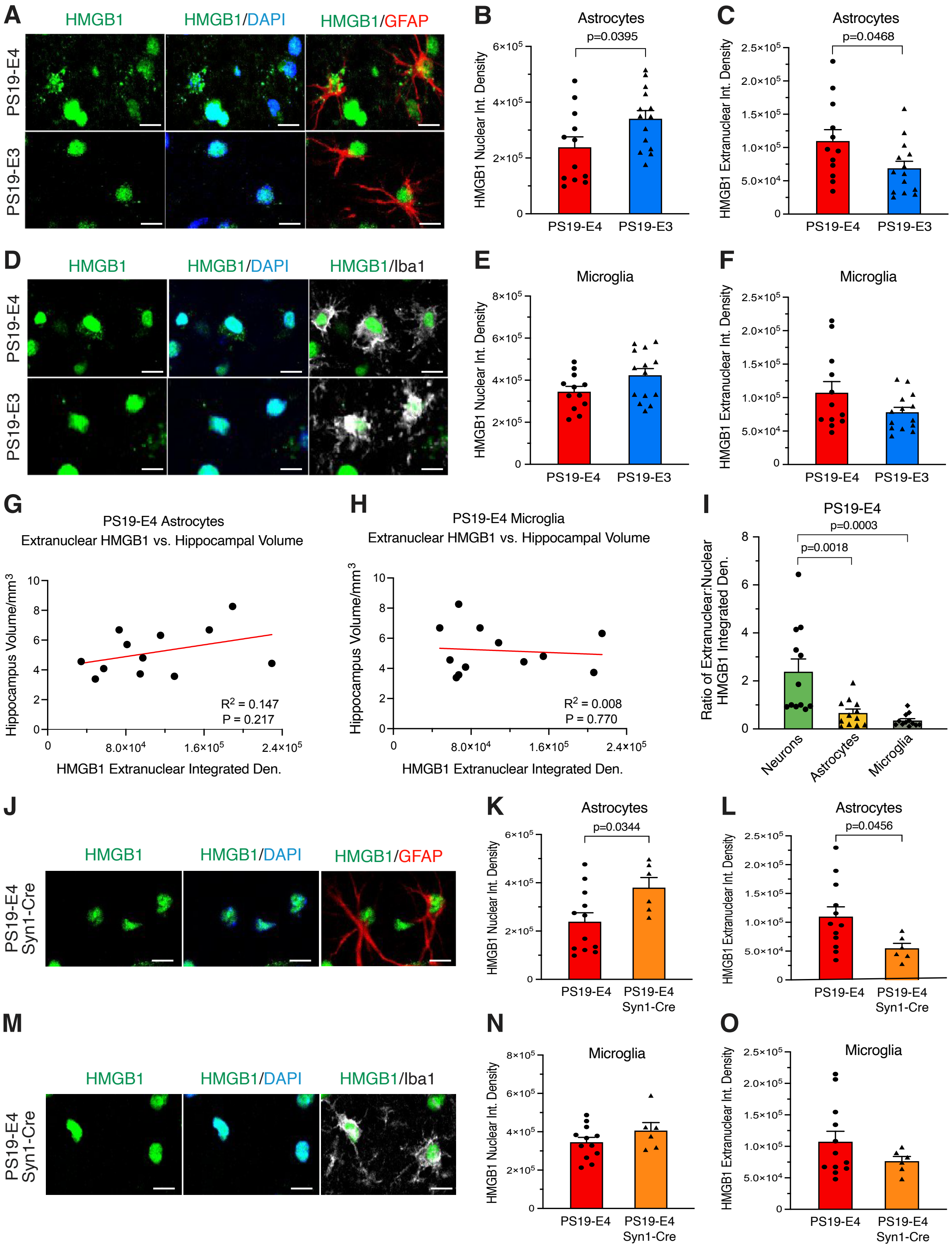

Figure S5. HMGB1 intracellular translocation also occurs in astrocytes, but not microglia, in aged PS19-E4 mice and removal of neuronal APOE4 reduces astrocytic HMGB1 translocation, Related to Figures 1 and 3.

(**A**) Representative images of immunostaining with anti-HMGB1, anti-GFAP, and DAPI in the dentate gyrus of the hippocampus (scale bar, 5 μm).

(**B**) Quantification of the nuclear integrated density of HMGB1 immunostaining in hippocampal astrocytes.

(**C**) Quantification of the extranuclear integrated density of HMGB1 immunostaining in hippocampal astrocytes.

(**D**) Representative images of immunostaining with anti-HMGB1, anti-Iba1, and DAPI in the dentate gyrus of the hippocampus (scale bar, 5 μm).

(**E**) Quantification of the nuclear integrated density of HMGB1 immunostaining in hippocampal microglia.

(**F**) Quantification of the extranuclear integrated density of HMGB1 immunostaining in hippocampal microglia.

(**G**) Correlation between the relative amounts of HMGB1 extranuclear density in astrocytes and hippocampal volume.

(**H**) Correlation between the relative amounts of HMGB1 extranuclear density in microglia and hippocampal volume.

(**I**) Quantification of the ratio of extranuclear:nuclear integrated density of HMGB1 immunostaining in hippocampal neurons, astrocytes, and microglia.

(**J**) Representative images of immunostaining with anti-HMGB1, anti-GFAP, and DAPI in the dentate gyrus of the hippocampus (scale bar, 5 μm).

(**K**) Quantification of the nuclear integrated density of HMGB1 immunostaining in hippocampal astrocytes.

(**L**) Quantification of the extranuclear integrated density of HMGB1 immunostaining in hippocampal astrocytes.

(**M**) Representative images of immunostaining with anti-HMGB1, anti-Iba1, and DAPI in the dentate gyrus of the hippocampus (scale bar, 5 μm).

(**N**) Quantification of the nuclear integrated density of HMGB1 immunostaining in hippocampal microglia.

(**O**) Quantification of the extranuclear integrated density of HMGB1 immunostaining in hippocampal microglia.

For all representative images and quantified data, mice were 10-months-old and belonged to either the PS19-E4, PS19-E3, or PS19-E4/Syn1-Cre groups as indicated. Quantified data in B,C,E,F,I (PS19-E4, n=12; PS19-E3, n=14) and K,L,N,O (PS19-E4, n=6; PS19-E4/Syn1-Cre, n=6) are represented as mean±SEM, unpaired two-sided t test. Data in G,H (PS19-E4, n=12) are Pearson's correlation analysis (two-sided).

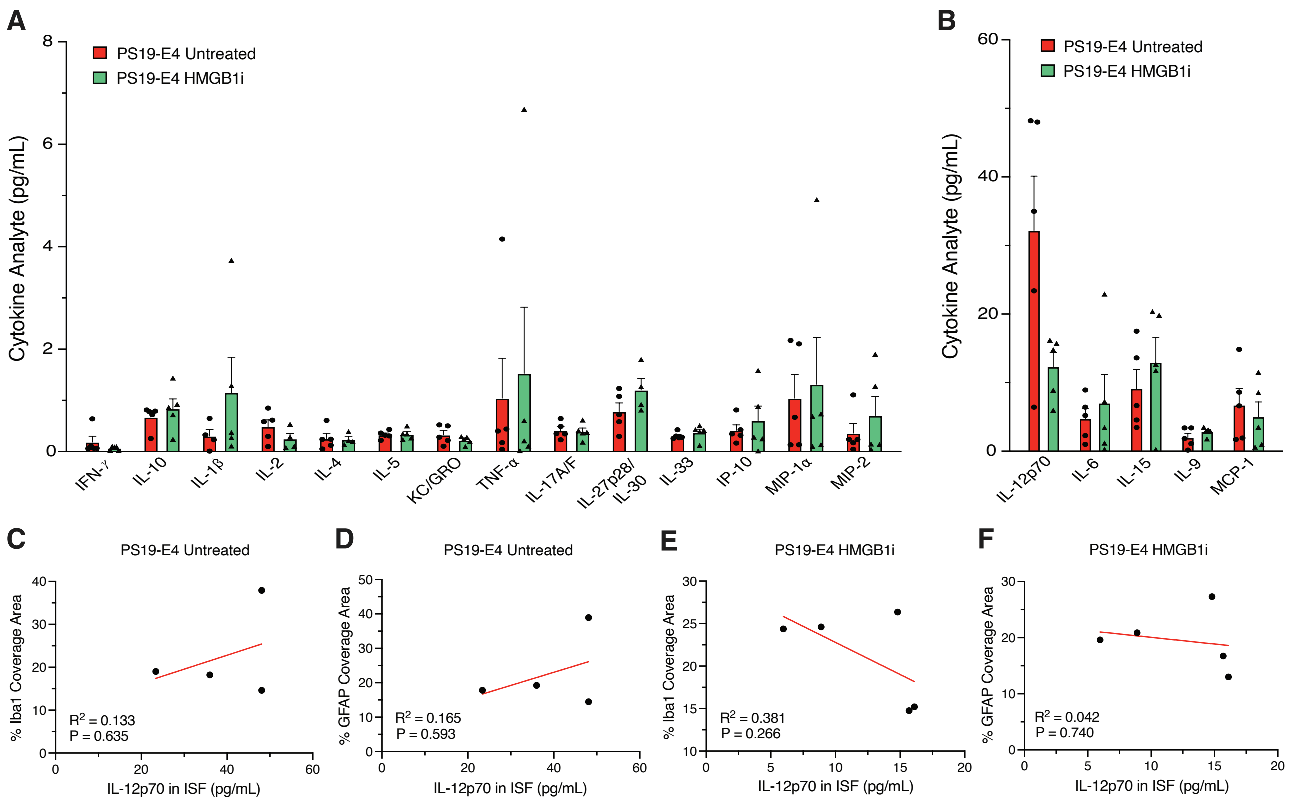

Figure S6. Treatment of PS19-E4 mice with HMGB1 inhibitors does not lead to significant alterations in the levels of other inflammatory cytokines in the ISF, Related to Figure 4.

(**A and B**) Cytokine protein levels measured by multiplex cytokine assay in the hippocampal interstitial fluid (ISF) of untreated or treated PS19-E4 mice.

(**C**) Correlation between the amount of secreted IL-12p70 and the percent coverage area of Iba1 microglia in untreated PS19-E4 mice.

(**D**) Correlation between the amount of secreted IL-12p70 and the percent coverage area of GFAP astrocytes in untreated PS19-E4 mice.

(**E**) Correlation between the amount of secreted IL-12p70 and the percent coverage area of Iba1 microglia in treated PS19-E4 mice.

(**F**) Correlation between the amount of secreted IL-12p70 and the percent coverage area of GFAP astrocytes in treated PS19-E4 mice.

All data were from 9.7-month-old PS19-E4 mice. Quantified data in A,B (PS19-E4 untreated, n=6; PS19-E4 HMGB1-In, n=5) are represented as mean±SEM, unpaired two-sided t test. Data in C,D (untreated PS19-E4, n=4; treated PS19-E4, n=5) are Pearson's correlation analysis (two-sided).

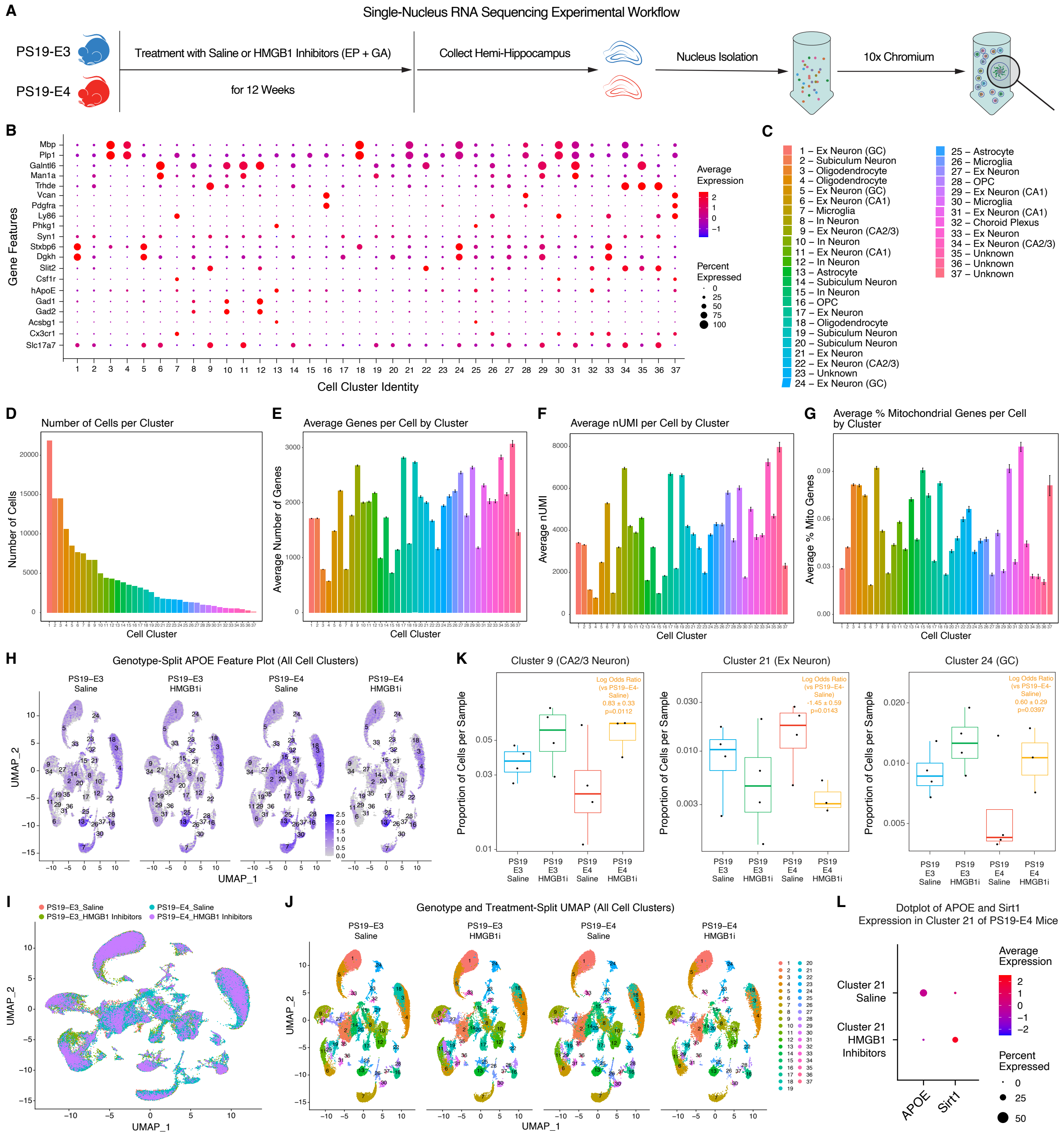

Figure S7. Quality control measures in snRNA-seq analysis of PS19-E4 and PS19-E3 mice without or with long-term HMGB1 inhibitor treatment, Related to Figure 7.

(**A**) Schematic of the single-nucleus RNA-sequencing workflow.

(**B**) Dot-plot illustrating the normalized average expression of selected marker genes for all 37 hippocampal cell clusters.

(**C**) Cell cluster identity of all 37 identified cell types.

(**D**) Number of cells per hippocampal cell cluster.

(**E**) Average genes per cell in each hippocampal cell cluster (± SEM).

(**F**) Average nUMI per cell in each hippocampal cell cluster (± SEM).

(**G**) Average percent mitochondrial genes per cell in each hippocampal cell cluster (± SEM).

(**H**) Feature plot illustrating the relative levels of normalized human *APOE* gene expression across all hippocampal cell clusters for each mouse genotype group and condition.

(**I**) UMAP plot of all cell clusters, highlighting the genotype and treatment groups with different colors.

(**J**) Genotype and treatment split UMAP plot highlighting all hippocampal cell clusters.

(**K**) Boxplot of the proportion of cells from each sample in cell clusters 9, 21, and 24. The log odds ratios are the mean ± standard error estimates of log odds ratio for clusters 79, 21, and 24, which represents the change in the log odds of cells per sample from HMGB1 inhibitor-treated PS19-E4 mice or saline-treated PS19-E3 mice belonging to the respective clusters compared to the log odds of cells per sample from saline-treated PS19-E4 mice.

(**L**) Dot-plot of normalized average expression of APOE and Sirt1 in excitatory neurons cell cluster 21 comparing HMGB1 inhibitor-treated or saline-treated PS19-E4 mice.

For details, also see Tables S2 and S3.

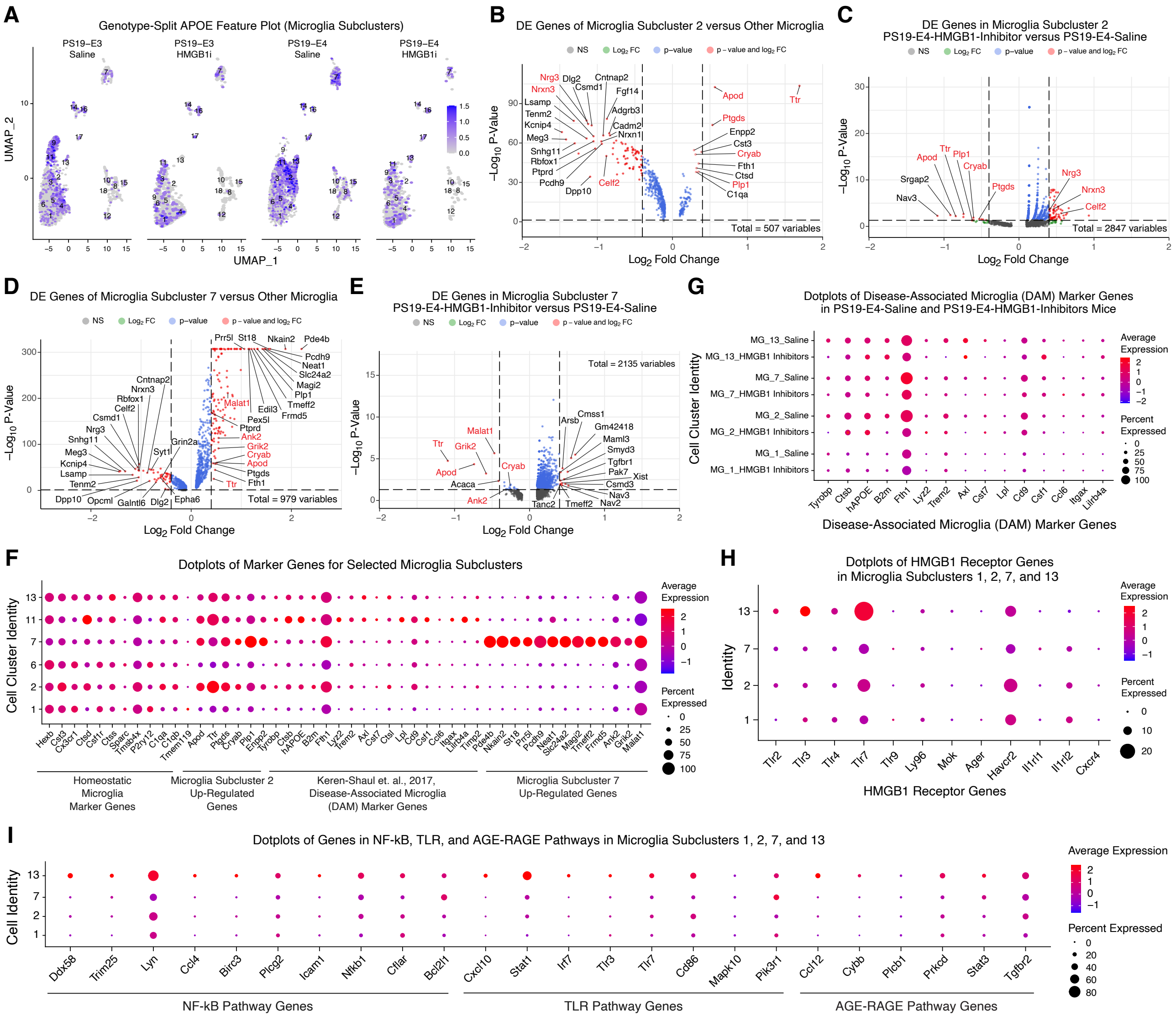

Figure S8. Long-term treatment of PS19-E4 mice with HMGB1 inhibitors diminishes disease-associated and enriches disease-protective subpopulations of microglia, Related to Figure 7.

(**A**) Feature plot illustrating the relative levels of normalized human *APOE* gene expression across all microglial cell subclusters for each mouse genotype group and condition.

(**B**) Volcano plots of the differentially expressed (DE) genes in microglial subcluster 2 versus other microglial subclusters.

(**C**) Volcano plot of the DE genes in microglial subcluster 2 in HMGB1 inhibitor-treated PS19-E4 mice versus saline-treated PS19-E4 mice.

(**D**) Volcano plots of the DE genes in microglial subcluster 7 versus other microglial subclusters.

(**E**) Volcano plot of the DE genes in microglial subcluster 7 in HMGB1 inhibitor-treated PS19-E4 mice versus saline-treated PS19-E4 mice.

(**F**) Dot-plot of normalized average expression of marker genes for selected microglial subclusters.

(**G**) Dot-plot of normalized average expression of marker genes for selected microglial subclusters, highlighting genes that are specifically upregulated in microglial subclusters 1, 2, 7, and 13 relative to other microglial subclusters in saline-treated versus HMGB1 inhibitor-treated PS19-E4 mice.

(**H**) Dot-plot of normalized average expression of HMGB1 receptor genes in microglial subclusters 1, 2, 7, and 13.

(**I**) Dot-plot of normalized average expression of genes related to NF-kB, TLR, and AGE-RAGE pathways in microglial subclusters 1, 2, 7, and 13.

For volcano plots in B-E, the dashed lines represent log2-fold-change threshold of 0.4 and p-value threshold of 0.05. Gene names highlighted in red text indicate they are selected marker genes of this microglial subcluster.

For details, also see Tables S4 and S5.

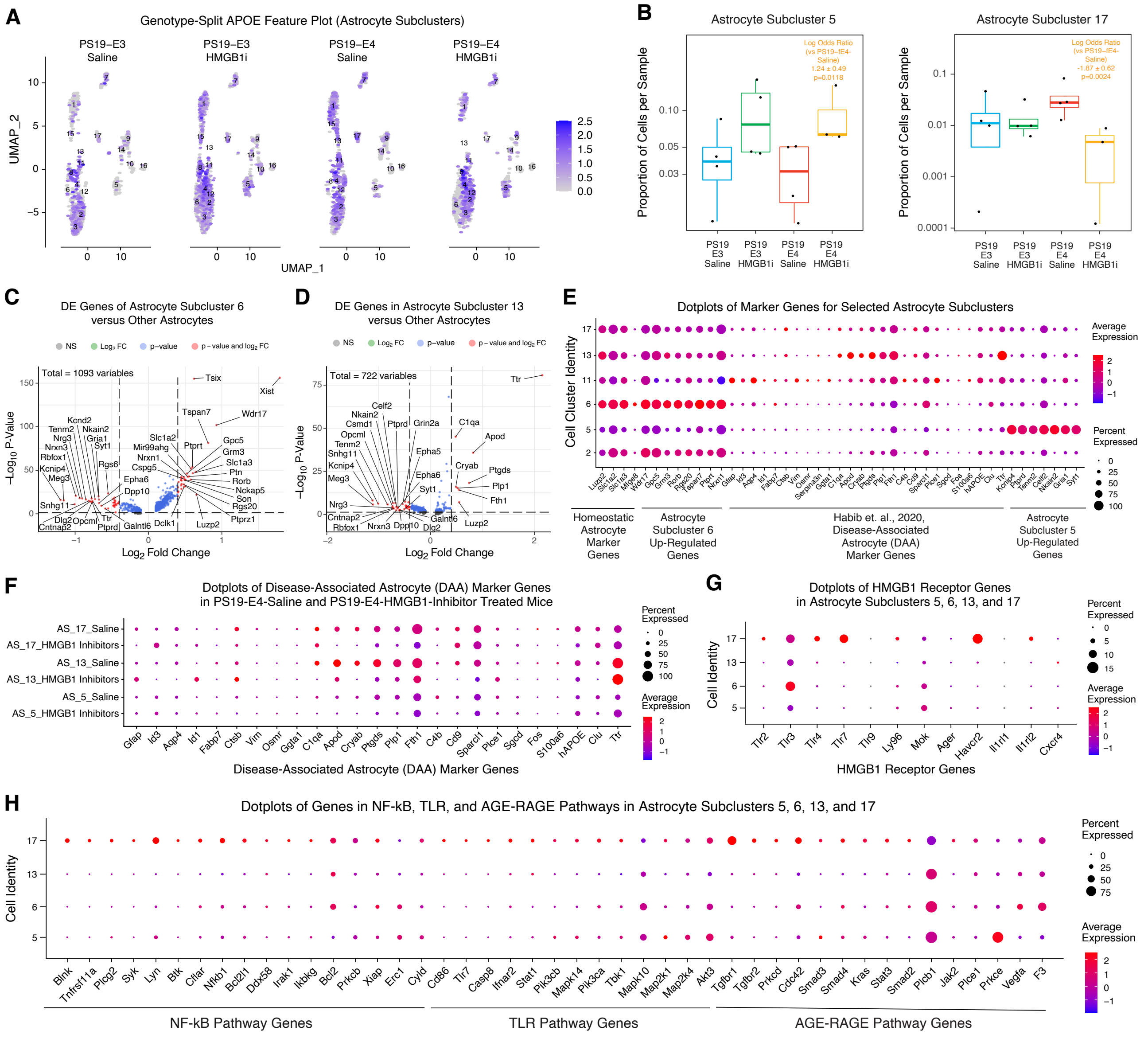

Figure S9. Long-term treatment of PS19-E4 mice with HMGB1 inhibitors diminishes disease-associated and enriches disease-protective subpopulations of astrocytes, Related to Figure 7.

(**A**) Feature plot illustrating the relative levels of normalized human *APOE* gene expression across all astrocyte cell subclusters for each mouse genotype group and condition.

(**B**) Boxplot of the proportion of cells from each sample in astrocyte subclusters 5 and 17. The log odds ratios are the mean ± standard error estimates of log odds ratio for clusters 5 and 17, which represents the change in the log odds of cells per sample from HMGB1 inhibitor-treated PS19-E4 mice or saline-treated PS19-E3 mice belonging to the respective clusters compared to the log odds of cells per sample from saline-treated PS19-E4 mice.

(**C**) Volcano plots of the differentially expressed (DE) genes in astrocyte subcluster 6 versus other astrocyte subclusters.

(**D**) Volcano plots of the DE genes in astrocyte subcluster 13 versus other astrocyte subclusters.

(**E**) Dot-plot of normalized average expression of marker genes for selected astrocyte subclusters.

(**F**) Dot-plot of normalized average expression of marker genes for selected astrocyte subclusters, highlighting genes that are specifically upregulated in astrocyte subclusters 5, 13, and 17 relative to other astrocyte subclusters in saline-treated versus HMGB1 inhibitor-treated PS19-E4 mice.

(**G**) Dot-plot of normalized average expression of HMGB1 receptor genes in astrocyte subclusters 5, 6, 13 and 17.

(**H**) Dot-plot of normalized average expression of genes related to NF-kB, TLR, and AGE-RAGE pathways in astrocyte subclusters 5, 6, 13, and 17.

For volcano plots in C,D, the dashed lines represent log2-fold-change threshold of 0.4 and p-value threshold of 0.05.

For details, also see Tables S6 and S7.

Table S1. Demographic information of samples used in Figure 1I–1K, Related to Figure 1.

Sample ID	Study	APOE Genotype	Cogdx	Age at Death	Msex	Ceradsc
840	ROS	4/4	4	82.7	0	1
866	ROS	4/4	4	83.6	0	1
705	ROS	4/4	4	83.5	1	1
033	ROS	4/4	4	88.5	1	2
327	ROS	3/3	4	89.7	1	1
206	ROS	3/3	4	80.2	1	4
554	ROS	3/3	4	92.0	0	2
901	ROS	3/3	4	92.7	0	2

The demographic and basic pathological information of the 8 sample from the ROSMAP cohort. Ceradsc, cerad score; Cogdx, cognitive diagnosis; Msex, male sex.
